# Dynamic Trk and G Protein Signalings Regulate Dopaminergic Neurodifferentiation in Human Trophoblast Stem Cells

**DOI:** 10.1371/journal.pone.0143852

**Published:** 2015-11-25

**Authors:** Eing-Mei Tsai, Yu-Chih Wang, Tony Tung-Yin Lee, Cheng-Fang Tsai, Hung-Sheng Chen, Feng-Jie Lai, Kazunari K. Yokoyama, Tsung-Hsun Hsieh, Ruey-Meei Wu, Jau-nan Lee

**Affiliations:** 1 Department of Obstetrics and Gynecology, Kaohsiung Medical University Hospital and Center of Excellence for Environmental Medicine, Kaohsiung Medical University, Kaohsiung 807, Taiwan; 2 Accelerated Biosciences Corp., Manhattan Beach, California 90266, United States of America; 3 Graduate Institute of Medicine, Kaohsiung Medical University, Kaohsiung 807, Taiwan; 4 Department of Dermatology, Chi-Mei Medical Center, Tainan 710, Taiwan; 5 Department of Physical Therapy and Graduate Institute of Rehabilitation Science, College of Medicine, Chang Gung University, Taoyuan City 33302, Taiwan; 6 Department of Neurology, National Taiwan University Hospital, Taipei 10048, Taiwan; Temple University School of Medicine, UNITED STATES

## Abstract

Understanding the mechanisms in the generation of neural stem cells from pluripotent stem cells is a fundamental step towards successful management of neurodegenerative diseases in translational medicine. Albeit *all-trans* retinoic acid (RA) has been associated with axon outgrowth and nerve regeneration, the maintenance of differentiated neurons, the association with degenerative disease like Parkinson's disease, and its regulatory molecular mechanism from pluripotent stem cells to neural stem cells remain fragmented. We have previously reported that RA is capable of differentiation of human trophoblast stem cells to dopamine (DA) committed progenitor cells. Intracranial implantation of such neural progenitor cells into the 6-OHDA-lesioned substantia nigra pars compacta successfully regenerates dopaminergic neurons and integrity of the nigrostriatal pathway, ameliorating the behavioral deficits in the Parkinson’s disease rat model. Here, we demonstrated a dynamic molecular network in systematic analysis by addressing spatiotemporal molecular expression, intracellular protein-protein interaction and inhibition, imaging study, and genetic expression to explore the regulatory mechanisms of RA induction in the differentiation of human trophoblast stem cells to DA committed progenitor cells. We focused on the tyrosine receptor kinase (Trk), G proteins, canonical Wnt2B/β-catenin, genomic and non-genomic RA signaling transductions with *Tyrosine hydroxylase* (*TH*) gene expression as the differentiation endpoint. We found that at the early stage, integration of TrkA and G protein signalings aims for axonogenesis and morphogenesis, involving the novel RXRα/Gα_q/11_ and RARβ/Gβ signaling pathways. While at the later stage, five distinct signaling pathways together with epigenetic histone modifications emerged to regulate expression of TH, a precursor of dopamine. RA induction generated DA committed progenitor cells in one day. Our results provided substantial mechanistic evidence that human trophoblast stem cell-derived neural stem cells can potentially be used for neurobiological study, drug discovery, and as an alternative source of cell-based therapy in neurodegenerative diseases like Parkinson’s disease.

## Introduction

Pluripotent stem cells such as human embryonic stem (hES) cells and induced pluripotent stem (iPS) cells contain plasticity to generate NSCs, expressing signature features of neuroepithelia, neuronal morphology and functionality for disease modeling and therapeutic purposes with potential capacity for clinical management of Parkinson’s disease (PD) [1–4]. Furthermore, we have previously reported that *all-trans* retinoic acid (RA) is capable of differentiation of human trophoblast stem (hTS) cells to DA committed progenitor cells [5]. Intracranial implantation of such NSCs into the 6-OHDA-induced and -lesioned substantia nigra pars compacta successfully regenerates dopaminergic (DA) neurons and integrity of the nigrostriatal pathway, ameliorating the behavioral deficits in the PD rat model. However, the molecular mechanisms that drive the stem cells to NSCs remain incompletely understood and understanding of those regulatory mechanisms is essential and required before any pluripotent stem cells can be ultimately applied to translational medicine

RA, a derivative of vitamin A, is able to enter the nucleus to bind directly to target genes via nuclear receptors that elicit a well-known genomic RA signaling pathway or may crosstalk with other molecules to perform the non-genomic RA pathway in the regulation of neurodifferentiation, motor axon outgrowth and neural patterning [6–7]. It has been shown that extrinsic signal RA and intrinsic transcription factors Neurogenin2 (Ngn2) collaboratively trigger transcriptionally active chromatin in spinal motor neuron genes that determine specific cell fate during development [8], suggesting that the neural progenitors can integrate both cues and orchestrate chromatin changes for neuronal specification.

Neurotrophins belong to a family of growth factors, including nerve growth factor (NGF), brain-derived neurotrophic factor (BDNF), and neurotrophins 3 (NT3) and NT4. At the cell membrane, NGF binds to tyrosine kinase receptor (Trk) A (TrkA), while BDNF and NT4 bind to TrkB but NT3 binds to TrkC. Trk signaling is critical for cell survival and death events, regulating neuronal proliferation and differentiation, axonal growth and synaptic modulation and the assembly of cytoskeletal proteins into axons [9–11]. Neurotrophic factors also protect DA neurons and enhance their regeneration in PD [12].

G protein-coupled receptors (GPCRs) constitute a large protein family of receptors, which can transduce extracellular signals into intracellular target molecules via dissociation of Gαβγ protein complex into subunits Gα and Gβ that give rise to a variety of signal cascades, promoting survival and other functional regulation of neurons [13]. For example, Gα regulates differentiation and self-renewal in the developing NSCs [14] and affects the balance of attractive versus repulsive cues in the growth cone to regulate axonal pathfinding [15].

Here, we demonstrated that RA induced interplay between TrkA and G protein signaling pathways to initially involve in the axonogenesis, cell adhesions, and morphogenesis. Subsequently, RA induced a coordination of *TH* gene transcriptions and epigenetic histone modifications to generate DA committed progenitor cells, providing substantial evidence of the proof-of-concept that hTS cells can be a potential and reliable source for cell-based therapy in translational medicine [5].

## Materials and Methods

### Ethics statement

h TS cell line is a *de novo* cell line established in the same laboratory at Kaohsiung Medical University Hospital (KMUH) and published in a previous study [5]. The Institutional Review Board (IRB) on Human Subjects Research and Ethics Committees of KMUH approved this study and tissue donor informed consent procedure (KMUH-IRB-950140). Tissue donor participants provided written informed consent. The original IRB approval and written informed consent documents are recorded and filed in KMUH IRB documentation files with copies in my office. The National Cheng Kung University (NCKU) Institutional Animal Care and Use Committee (IACUC) specifically approved these animal experiments (IACUC Approval No. 98010). The NCKU IACUC guidelines aim to treat rats humanely and reduce animal suffering by use of anesthesia and analgesics, and provide nutritional and fluid support as described previously [9].

### Cell Culture and Differentiation

hTS cells were cultured in conditioned *α*-MEM plus 10% FBS at 37°C in 5% CO_2_ as described previously [5]. For neural differentiation, cells were treated by freshly prepared RA (10 μM, Sigma-Aldrich).

### Western Blot, Immunoprecipitation (IP), RNA Interference, and Flow Cytometry

These methods used were aimed to identify the molecular expression, protein-protein interaction and inhibition as described previously [5]. Specifically, plasmid construct used in this study yields over 95% of transfection rate. Furthermore, small interfering RNA (siRNA) and short hairpin RNA (shRNA) were purchased from the National RNAi Core Facility Platform, Institute of Molecular Biology/Genomic Research Center, Academia Sinica, Taipei, Taiwan. Data from flow cytometry were analyzed by Cell-Quest software (BD Biosciences). All reagents, antibodies and primers used were listed in S1–S4 Tables.

### Immunocytochemistry, TissueQuest Analysis, Double Immunogold Electron Microscopy and Live Cell Imaging and Calcium Measurements

These methods were performed for imaging studies. Briefly, cells were prepared as described previously [5]. For immunocytochemistry, cells were incubated with specific primary antibody in PBS at 4°C overnight, followed by adding appropriate fluorescent reagent-conjugated secondary antibody and DAPI staining to be able to observe by immunofluoresence microscope. For TissueQuest analysis, monoclonal antibodies against TH and CREB1 were used. Samples were observed by Zeiss AxioImager Z1 microscope and analyzed by TissueFaxs software. All data were analyzed by two technicians independently. For double immunogold electron microscopy, cells were incubated primary IgG antibody against RXRα, followed by probing with a secondary anti-mouse IgG 6 nm gold particles or anti-rabbit IgG 20 nm gold particles. Imaging was observed under Hitachi H-700 model transmission electron microscope. The detail procedures were described in Supporting Information.

### Quantitative PCR (qPCR) and ChIP-qPCR Analysis

These methods were applied to measure the expression of mRNAs and genes, performed by commercial kits according to the instructions. Detail information was described previously [5] and in Supplementary Materials.

### Statistics

Data obtained from qPCR, ChIP-qPCR, Western blot and flow cytometric analysis were calculated by Student’s t-test. Data express as mean ± SD. p-value < 0.05 indicate statistically significant. The predictive ability of immunofluorescence intensity was judged by the R-square in the immunofluorescence TissueQuest analysis.

## Results

### 1. A Cohort of Morphogenetic Events at the Early Stage of Differentiation

**Fig 1:**
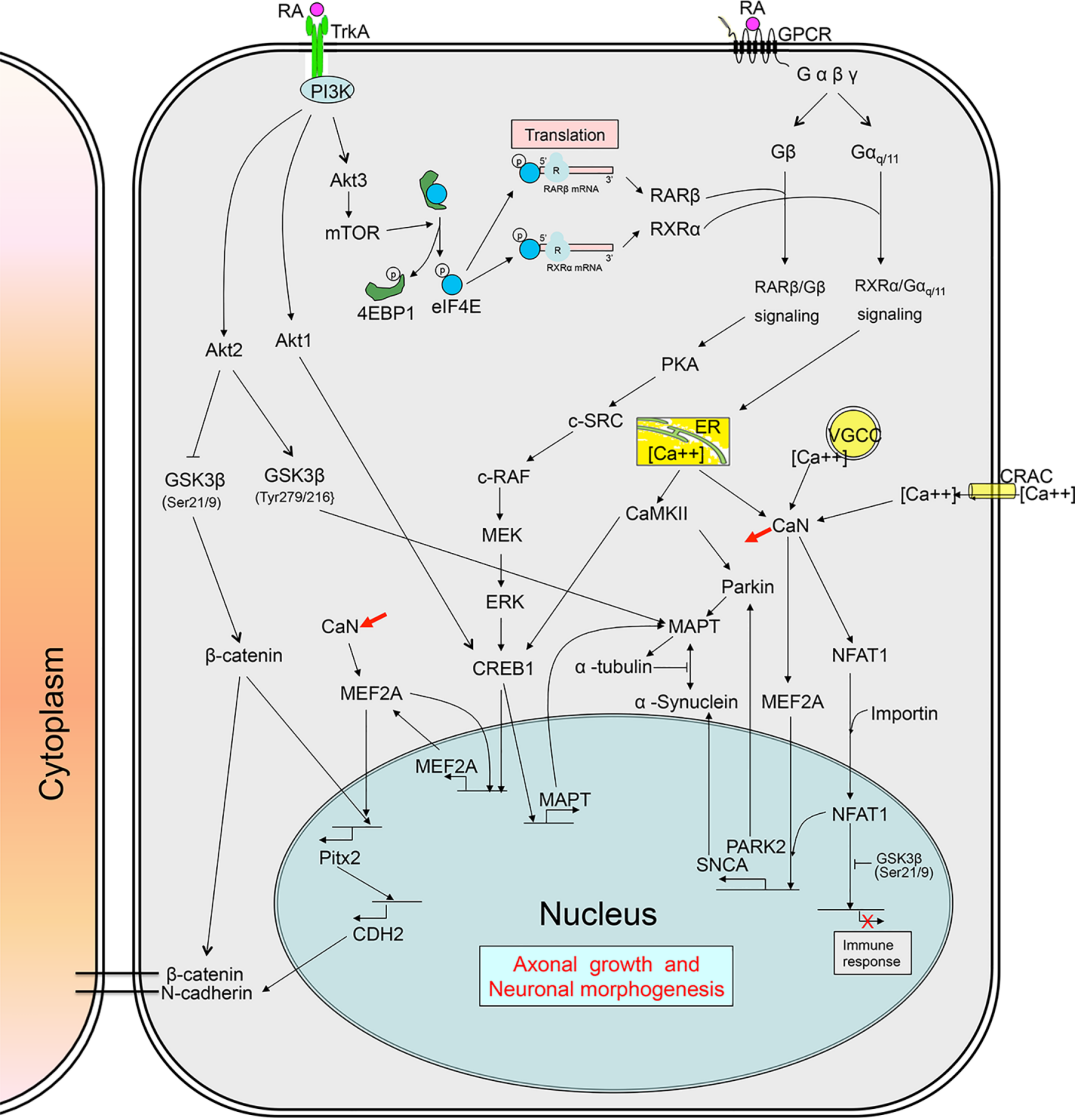
Schematic Illustration of Molecular Networks of RA Induction at the Early Stage of Differentiation in hTS Cells.

Fig 1 illustrates a molecular network of RA induction at 4 hr, by which we demonstrated the regulatory molecular mechanisms through the integration of TrkA and G protein signaling pathways that contributed to microtubule assembly and stabilization, axonal growth, cell-cell interaction and morphogenesis

#### RA induces subcellular localization of RARβ and RXRα via TrkA/Akt3/mTOR pathway

**Fig 2:**
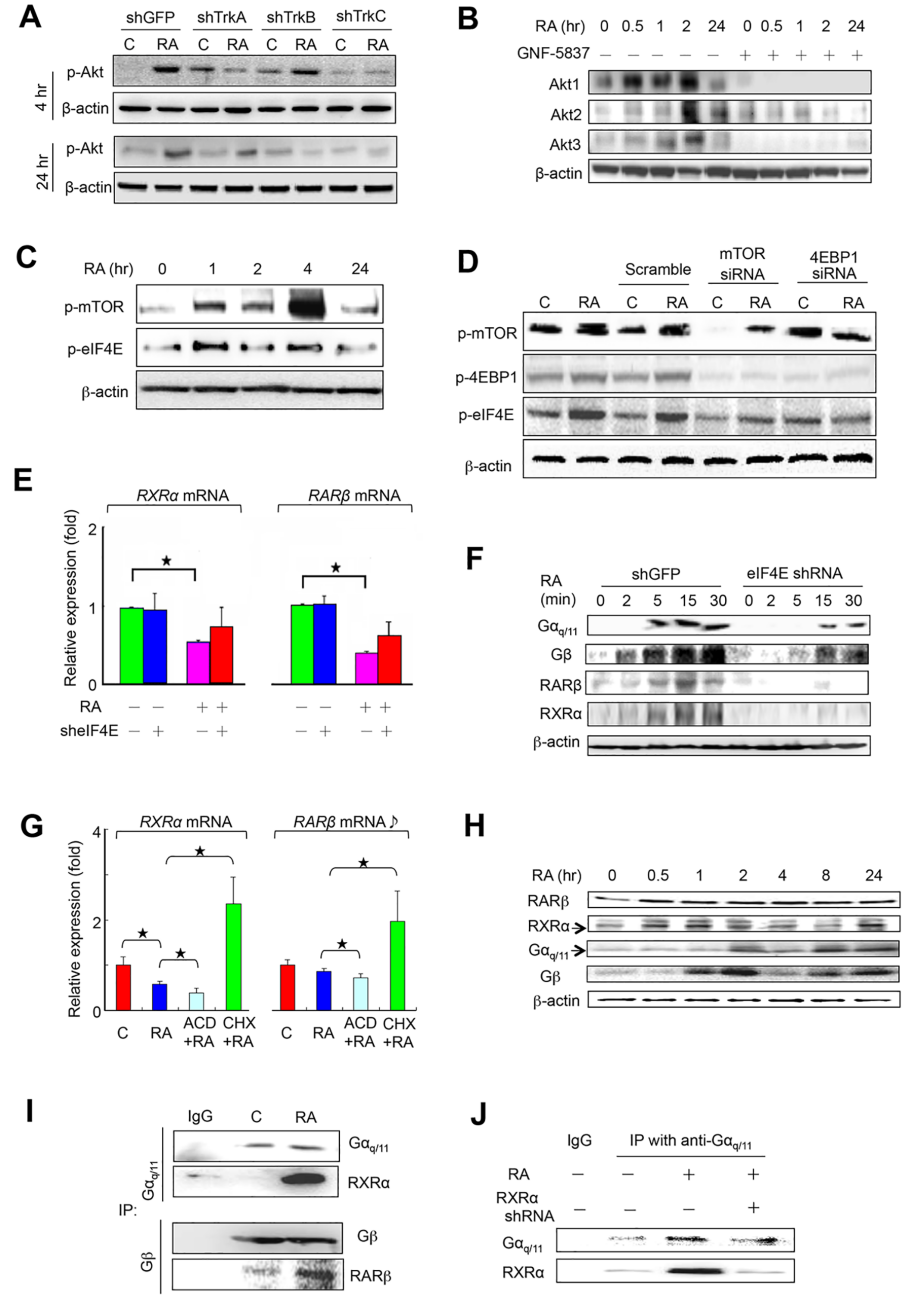
Subcellular Localization of RARβ and RXRα. (A-B) Knockdown of TrkA and TrkB reduced Akt1 and Akt3 expressions at 4 hr (upper) and 24 hr (lower panel), respectively, evidenced by inhibitory GNF-5837 by immunoblotting assays. β-actin as loading control. (C) RA activated mTOR and eIF4E at 4 hr, but not 24 hr. (D) Knockdown of mTOR resulted in reduction of phosphorylation of 4EBP1, but not eIF4E, and knockdown of 4EBP1 did not reduce eIF4E. (E) RA reduced both RXRα mRNA (left) and RARβ mRNA (right) levels at 2 min by qPCR, but did not change by using eIF4E shRNA. Data represent mean ± SD, n = 6, ^★^:p < 0.05. (F) RA-induced expressions of Gα_q/11_, Gβ, RARβ and RXRα in a timeline and their responses by knockdown of eIF4E shRNA. shGFP as a positive control. (G) RA-reduced RXRα mRNA and RARβ mRNA levels were promoted by ACD (100 μg/ml), but not CHX (100 μg/ml) at 30 min by qPCR assay. Data represent mean ± SD, n = 4, ^★^:p < 0.05. C: control. (H) RA induced different expression patterns of RARβ, RXRα, Gβ and Gα_q/11_ by immunoblotting assay. (I) IP assay showed an interaction between Gβ and RARβ and also between Gα_q/11_ and RXRα while (J) knockdown of RXRα inhibited the latter.

To address the RA effect on Trk expression, we found that RA induced expression of TrkA, but not TrkB and TrkC, to promote PI3K/Akt pathways [16] by phosphorylation of three Akt subunits, thereby, establishing the TrkA/PI3K/Akt1, the TrkA/PI3K/Akt2, and the TrkA/PI3K/Akt3 signaling pathways (Fig 2A and 2B). These pathways were verified by the inhibitory action of TrkA shRNA (Fig 2A), Trk inhibitor GNF-5837 (Fig 2B), and PI3K inhibitor Wortmannin (S1A Fig). Interestingly, only Akt3, not Akt1 and Akt2, interacted with and resulted in the phosphorylation of the mammalian target of rapamycin (mTOR) (Fig 1 and S1B–S1D Fig). Phosphorylated mTOR then interacted with the mRNA translation repressor 4E-binding protein (4EBP1) resulting in the increase of 4EBP1 (Fig 2D and S1E Fig). In turn, 4EBP1 activation resulted in the phosphorylation of eukaryotic translation initiation factor 4E (elF4E) (Fig 2C and 2D) to prevent the assembly of elF4E into the cap-dependent translational inhibitor eIF4F complex, thereby, initiating the global cap-dependent translation [17]. This was supported by the rapid reduction of both RXRα mRNA and RARβ mRNA by qPCR analysis (Fig 2E) and knockdown of eIF4E reduced RXRα and RARβ levels, but not Gβ and Gα_q/11_, (Fig 2F). Both RXRα mRNA and RARβ mRNA expressions were inhibited by the transcription inhibitor actinomycin D (ACD), but not the translation inhibitor cycloheximide (CHX) (Fig 2G). These results suggested that RA induced local translation of RARβ and RXRα via activation of the TrkA/PI3K/Akt3/mTOR signaling, representing a novel non-genomic RA effect.

#### Integration of TrkA and G protein signalings generates non-genomic RARβ/Gβ and RXRα/Gα_q/11_ signaling pathways

**Fig 3:**
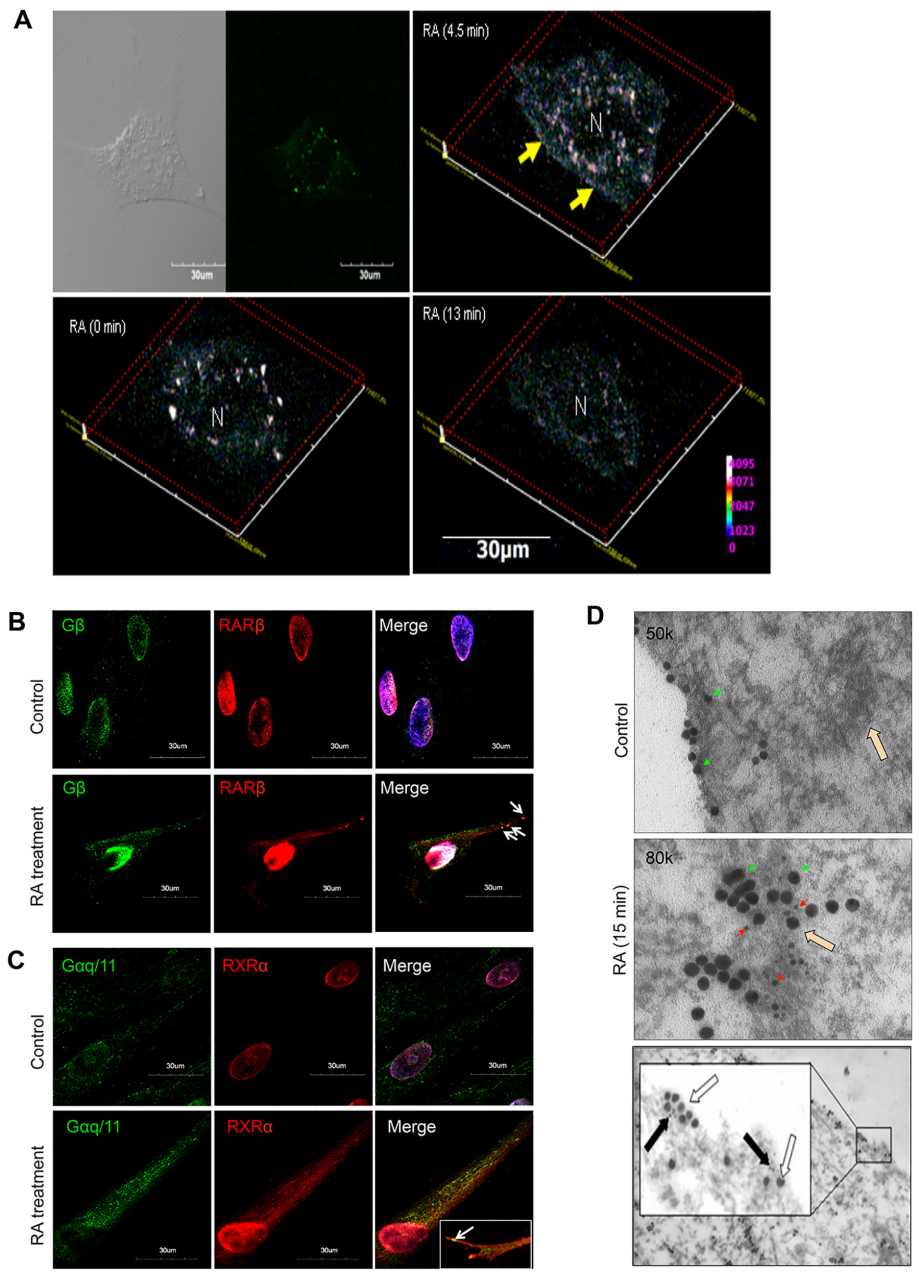
Imaging of RARβ/Gβ and RXRα/Gα_q/11_ Complexes. (A) Representative micrographs of real-time confocal immunofluorescence microscopy showing RA at 0 min, 4.5 min, and 13 min. Bar scale: 30μm. (B) The distribution of Gβ (green) and RARβ (red) before RA stimulation (upper); while co-localization of them at the extended axonal membrane after RA (white arrow) (lower) and (C) a similar expression was observed in between Gα_q/11_ (green) and RXRα (red). Bar scale: 30μm. (D) Representative electron micrographs showing: the subcellular large gold-tagged Gα_q/11_ (20 μm, green arrow) and ER areas (blank arrow) before RA stimulation (upper). Co-localization of the small gold-tagged RXRα (6 μm, red arrow) and the large gold-tagged Gα_q/11_ (green arrow) at the ER areas (blank arrow) (middle) and at the subcellular regions after RA stimulation (15 min; lower). N: nucleus, Bar scale: 1 μm.

Meantime, GPCR transduced RA signal to intracellular G protein complexes, leading to the segregation of subunits Gα_q/11_ and Gβ at the subcellular regions (Fig 2H). Unexpectedly, interaction between RARβ and Gβ and between RXRα and Gα_q/11_ occurred (Fig 2I), evidenced by knockdown of RXRα in preventing interaction of RXRα and Gα_q/11_, for example (Fig 2J). To further explore these unique events, we created the green fluorescent protein *(*GFP)-tagged RXRα, the small gold particle-tagged RXRα (6 μm in size) and the large gold particle-tagged Gα_q/11_ (20 μm) in order to observe the molecular behaviors by imaging analysis. Real-time confocal immunofluoresence microscopy revealed the movement of GFP-tagged RXRα to the subcellular regions in 0 min, 4.5 min, and 13 min of RA induction (Fig 3A). Co-localization of immunoreactive RARβ and Gβ (Fig 3B), as well as RXRα and Gα_q/11_ (Fig 3C), was observed at the tips of growing axon. Furthermore, double immunogold electron microscopy demonstrated an anchorage of the small gold particle-tagged RXRα together with the large gold particle-tagged Gα_q/11_ at either the endoplasmic reticulum (ER) or the subcellular regions (Fig 3D). We concluded that RA induced the integration of TrkA and G protein signaling pathways to create the functional RARβ/Gβ and RXRα/Gα_q/11_ complexes at the axonal regions.

**Fig 4:**
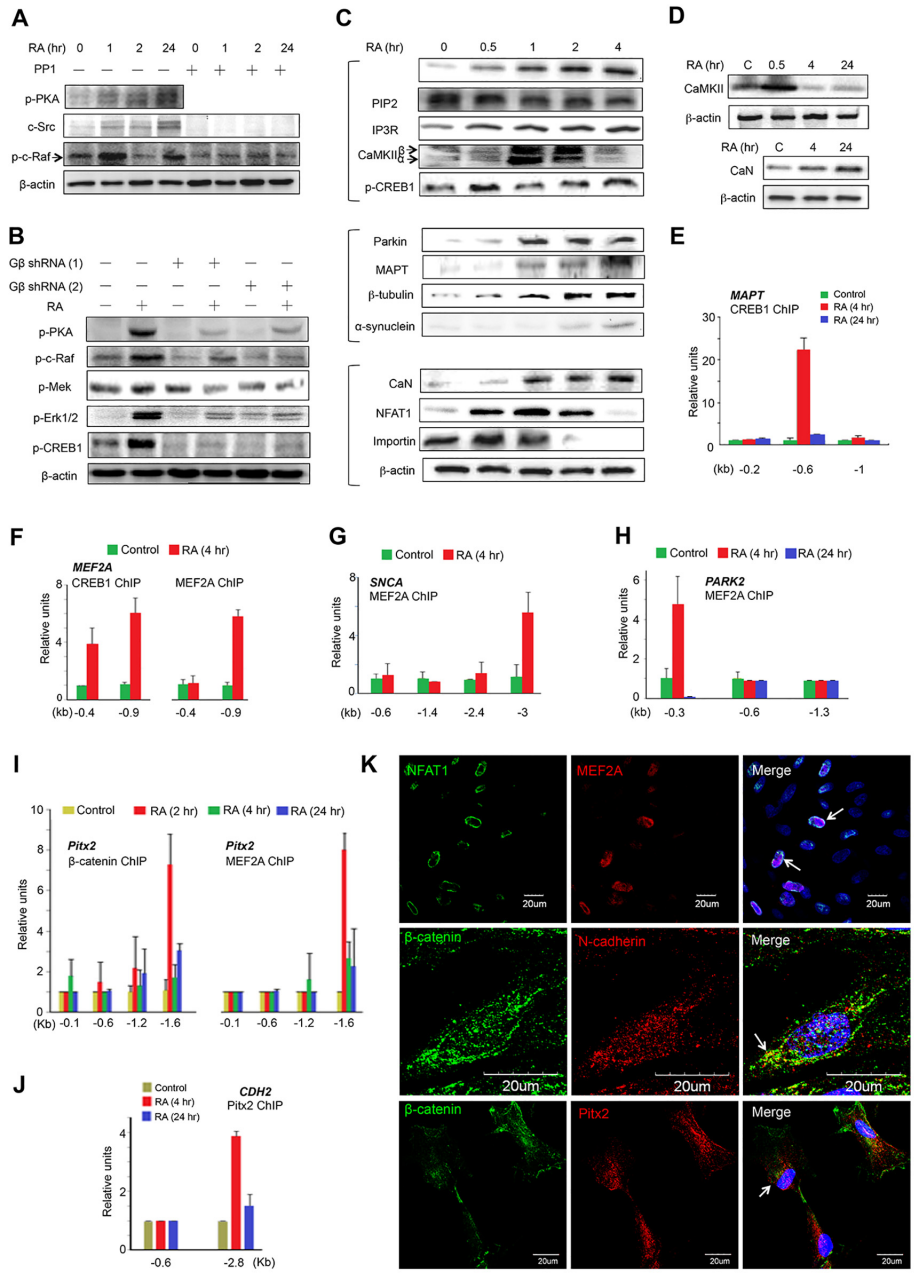
CREB1 and MAPT as Central Core Regulators in Axon Growth. (A-B) Linkage of PKA/c-Src/c-Raf pathway (A) and c-Raf/MEK/Erk1/2/CREB1 pathway (B) in a timeline by immunoblotting assays supported by PKA inhibitor PP1 and knockdown of Gβ. (C) RA activated the molecular components of Gα_q/11_/CaMKII/CREB1 pathway (upper), MAPT-centered complexes (middle) and Gα_q/11_/CaN/NFAT1 pathway (lower) at 4 hr induction. (D) Responses of CaMKII and CaN to RA at different time induction by immunoblotting assay. β-actin: loading control. (E-J) ChIP-qPCR analysis at different site of the promoter of genes by RA induction showing: (E) CREB1 transcribed *MAPT* gene at 4 hr, but not 24 hr; (F) CREB1 transcribed *MEF2A* gene (left) and MEF2A performed a transcriptional autoregulation (right) at 4 hr; (G-H) MEF2A transcribed *SNCA* gene at 4 hr (G) and *PARK2* gene at 4 hr, but not 24 hr (H); (I) β-catenin transcribed *Pitx2* gene at both 2 hr and 24 hr (left panel) and MEF2A transcribed *Pitx2* gene at 2 hr and (J) Pitx2 transcribed *CDH2* gene prominently at 4 hr, but not 24 hr. Data represent mean ± SD of quadruplicates. (K) Immunofluorescence imaging (4 hr) revealed that RA induced co-expression of NFAT1 and MEF2A (arrow; upper) and β-catenin and Pitx2 (arrow; middle) in the nucleus, while β-catenin and N-cadherin (arrow; lower) at the subcellular regions. Bar scale: 20μm.

Functionally, these complexes initiated two non-genomic RA signaling pathways independently. First, RARβ/Gβ signal activated its downstream effector protein kinase A (PKA) via phosphorylation and proto-oncogene c-Src, followed by a chain of proteins known as c-Raf/phospho(p)-MEK/p-Erk1/2 pathway (Fig 4A and 4B). Immediately, Erk1/2 activation resulted in phosphorylation of cAMP responsive element binding protein 1 (CREB1). This pathway was inhibited by c-Src inhibitor PP1, knockdown of Gβ (Fig 4A and 4B) and MAPK/Erk inhibitor PD98059 (S1F and S1G Fig). Therefore, a linkage between the RARβ/Gβ and the c-Src/c-Raf/p-MEK/p-Erk1/2/CREB1 pathway was established.

Second, RXRα/Gα_q/11_ signal, on the other side, catalyzed phosphatidylinositol 4,5-biphosphate (PIP2) into two-second messengers, including inositol 1,4,5-triphosphate (IP3) to activate its receptor IP3R at the endoplasmic reticulum (ER) (Fig 4C). Therefore, the intracellular calcium levels elevated and this action was blocked by IP3R inhibitor 2-aminoethoxydiphenyl borate (2-APB) (S2A Fig). The increased intracellular calcium activities rapidly induced a transient activation of calcium/calmodulin-dependent protein kinase II (CaMKII) within 2 hr to consequently phosphorylate CREB1 via interaction (Fig 4C and 4D and S2B Fig) and CREB1 expression was suppressed by CaMKII inhibitor KN93 (S1E Fig). An RXRα/Gα_q/11_/CaMKII/CREB1 pathway was formed.

On the other side, RA also induced activation of the TrkA/PI3K/Akt1 cascade (Fig 2A and 2B). Akt1 selectively caused the phosphorylation of its downstream CREB1 via direct interaction (S2C Fig) to form the TrkA/PI3K/Akt1/CREB1 pathway. We confirmed this pathway by the inhibitory action of PI3K inhibitor Wortmannin (S1A Fig) and Akt inhibitor MK2206 (S1C Fig). To this end, all three pathways converged at the CREB1 molecule to increase its nuclear localization.

#### CREB1 exhibits transcriptional capability to generate MEF2A and MAPT for microtubule assembly

In the nucleus, ChIP-qPCR analysis revealed that CREB1 induced two transcriptional effects: i) it targeted gene *MAPT* at 4 hr induction, but not at 24 hr, for *MAPT* transcription (Fig 4E), leading the migration of *MAPT* transcripts to the appropriate sites in axonal growth cones for further local synthesis of microtubule-associated protein tau (MAPT) and ii) it targeted gene *MEF2A* to produce more MEF2A to be further activated for biological functions (Fig 4F). These results suggest that CREB1 is a critical core regulator in axonogenesis. CREB1 is required for neuronal survival and axonal growth in neurogenesis [18–19].

#### MEF2A promotes axonogenesis via generation of α-Synuclein, Parkin, Pitx2, and N-cadherin

Unlike the transient expression of CaMKII, intracellular calcium elevation also induced expression of the calcium-dependent protein phosphatase calcineurin (CaN) and CaN expression became prominent as differentiation proceeded to 24 hr (Fig 4D). Active CaN activated MEF2A via dephosphorylation to form the RXRα/Gα_q/11_/CaN/MEF2A pathway (S3A Fig), resulting in nuclear localization of MEF2A [20]. In the nucleus, MEF2A exhibited several transcriptional activities by targeting: i) gene *MEF2A* in a model of transcriptional autoregulation circuitry (Fig 4F); ii) gene *SNCA* to produce a presynaptic neuronal protein α-Synuclein (Fig 4G and 4C), associated with the native synaptic vesicles, microtubule cytoskeletal function, phospholipids regulation and neurotransmitter release [21]; iii) gene *PARK2* to produce protein E3 ubiquitin ligase Parkin to subsequently form MAPT/Parkin complex (Fig 4C and 4H and S3B Fig). Mutations in *PARK2* gene causes autosomal recessive juvenile Parkinsonism [22], and iv) gene *Pitx2* to produce paired-like homeodomain transcription factor 2 (Pitx2) (Fig 4I). In turn, Pitx2 transcribed gene *CHD2* (Fig 4J) to ultimately produce a calcium dependent cell-cell adhesion glycoprotein N-cadherin. We concluded that an integration of TrkA and G-protein signaling pathways is involved in the production of α-Synuclein, Parkin, Pitx2 and N-cadherin mediating MEF2A at the early neurodifferentiation.

#### Intracellular calcium signals regulate axonogenesis via stage-specific CaMKII/CaN distribution

Interestingly, the transient CaMKII interacted to and activated Parkin to form the Parkin/MAPT complexes (Fig 4C and S3B Fig), involving in the microtubule assembly and stabilization. Parkin may function to protect dopaminergic neurons from death [23]. Nevertheless, CaMKII activities reduced gradually and became absent in the later differentiation (between 4 and 24 hr) (Fig 4D). In contrast, CaN expressed weakly at the initial stage but its expression gradually increased up to the later stage (24 hr) of differentiation. This fact indicates the importance of intracellular calcium activities in the regulation of CaMKII and CaN expressions at the early axonogenesis. Indeed, in addition to the intracellular ER calcium releases, cells require the presence of other regulatory intracellular calcium mechanisms to maintain an appropriate CaMKII/CaN ratio for axonogenesis.

For example, hTS cells were cultured in calcium-free medium. Live cell imaging studies revealed that the RA-induced depletion of intracellular ER calcium could be compensated and rescued by adding extrinsic CaCl_2_ (S3C Fig), suggesting the presence of calcium release-activated calcium channels (CRACs). Furthermore, by adding extracellular KCl also rescued the intracellular calcium levels after total depletion of ER calcium releases (S3C Fig). Pretreatment of a VGCC antagonist nifedipine was able to abolish the KCl-induced intracellular calcium increases (S3C Fig), suggesting the involvement of L-type voltage-gated calcium channels (VGCCs). These results indicated that the maintenance of intracellular calcium balance may require the integration of ER, VGCCs, and CRACs. Therefore, the stage-specific difference of intracellular calcium levels would determine the CaMKII/CaN ratio that impacts on the cellular behaviors during the differentiation processes. An increased CaMKII activity, for example, activated locally encoded VGCC activities rather than to integrate Ca^2+^ flux, which increases intracellular calcium levels [24].

#### RXRα/Gα_q/11_/CaN pathway enhances NFAT1 role in regulating axonal extension

Furthermore, CaN activity resulted in dephosphorylation of its downstream transcription factor NFAT1 (Fig 4C), allowing the nuclear translocalization of NFAT1 carried by transporter importin (S1A and S1B Fig). Whereas NFAT1 was supposed to act on its related immune genes via interaction; however, this action was prevented by the rephosphorylation of GSK3β (S4C Fig) [25]. Alternatively, NFAT1 bound to MEF2A to be a co-activator of MEF2A in the nucleus (S4D Fig), expressing a co-localization immunocytochemically (Fig 4K). However, NFAT1 can be a regulator in the proliferation and differentiation of NSCs in the neural tube, whereas NFAT1 binds directly to Dvl that represses the canonical Wnt/β-catenin signaling pathway in chick embryo [26]. Therefore, CaN/NFAT1 signal may perform its role in regulating axon extension [27] and is required to induce neurite outgrowth because the lack of NFAT family causes complete loss of axonal extension [28]. The linkage of the RXRα/Gα_q/11_/ER calcium and CaN/NFAT1 cascades was supported by the inhibitory action of IP3R inhibitor 2-APB (S2D Fig) and NFAT1 siRNA (S4E Fig).

#### TrkA/Akt2/GSK3β cascades are involved in regulation of morphogenesis and axonal growth

**Fig 5:**
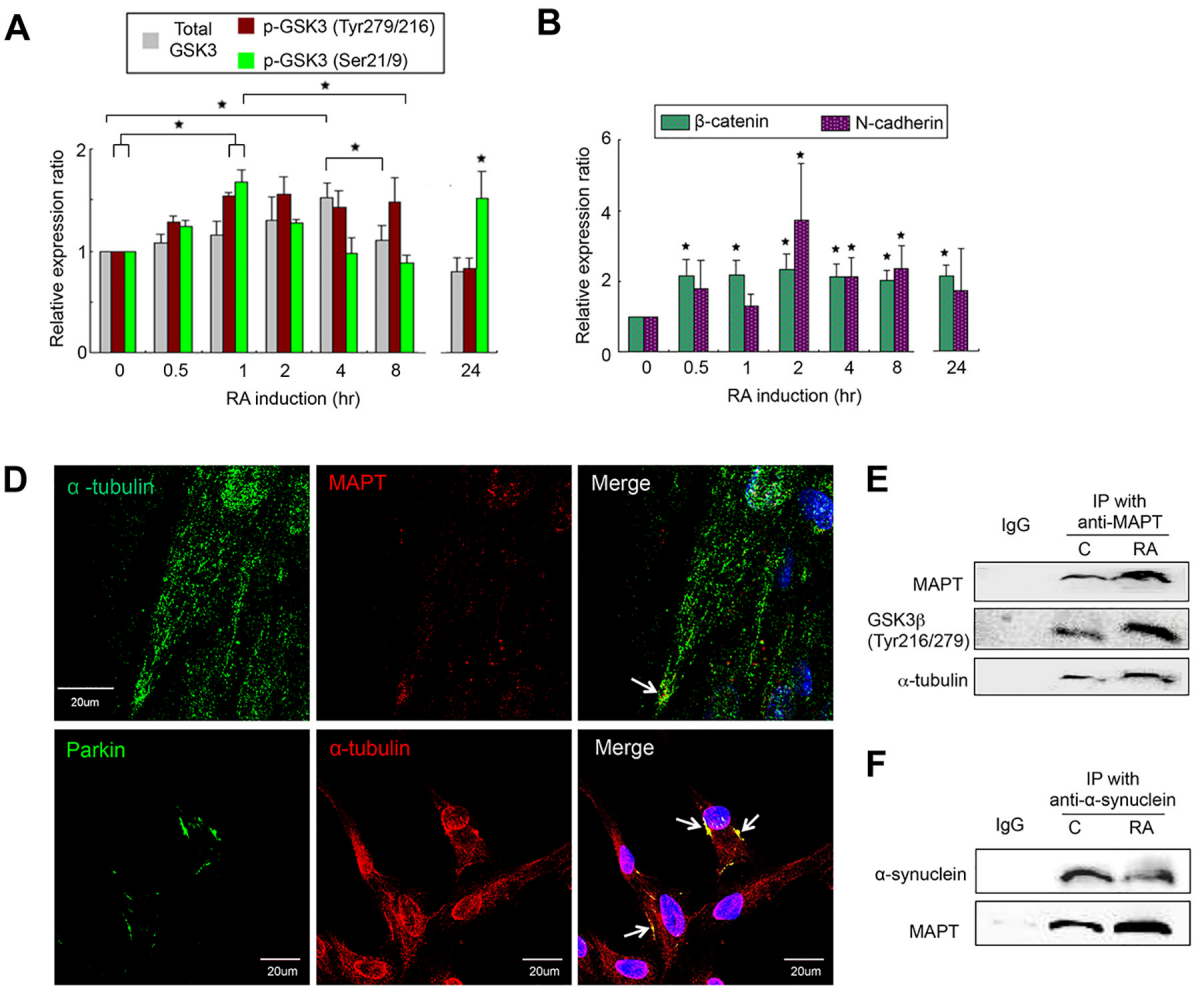
Multifaceted GSK3β in the Early Morphogenesis. (A-B) Dynamic expression of GSK3β by phosphorylation at Ser21/9 or Tyr279/216 sites (A) and of β-catenin and N-cadherin (B) after RA induction by immunoblotting assay. Data represent mean ± SD, n = 3, ^★^: p < 0.05. (C) Immunofluorescence imaging of RA induction (4 hr) showing: co-expressions of α-tubulin and MAPT at the axonal areas (arrow; upper); Parkin and α-tubulin at the subcellular regions (arrow; lower). (D-E) IP assays showing an interaction of MAPT to GSK3β-Tyr279/216 and α-tubulin (E) and to α-Synuclein (F). IgG as negative control, C as control.

Glycogen synthase kinase 3β (GSK3β) in response to extracellular neurotrophins is involved in cellular morphogenesis, depending on the phosphorylation site; for example, phosphorylation at Ser21/9 or Tyr279/216 may express divergent responses in the regulation of axonal growth [29–30]. In hTS cells, RA induced activation of the TrkA/PI3K/Akt2 cascades (Fig 2A and 2B) followed by phosphorylation of GSK3β at both Ser21/9 and Tyr279/216 sites via interaction at early 30 min of induction for different functions (Fig 5A and S5A Fig).

1) GSK3β-Ser21/9 signaling: An active GSK3β-Ser21/9 induced accumulation of β-catenin (Fig 5B) to also exhibit two actions: i) to form the β-catenin/N-cadherin complexes at the subcellular regions, showing co-localization immunocytochemically (Fig 4K), which guides the microtubule to the gap junction plaque at the cell-cell borders for cellular interaction [31] or involves in the initiation of myelination [32]; and ii) to enter the nucleus (S5B Fig) where it recruited lymphoid enhancer-binding factor 1 (LEF1) by direct interaction (S5C Fig), which in accompany with CaN/MEF2A signaled to target gene *Pitx2* for transcription (Fig 4l). A co-localization of β-catenin and Pitx2 was observed immunocytochemically (Fig 4K). In turn, Pitx2 targeted gene *CDH2* to produce N-cadherin (Fig 4J). Therefore, β-catenin, N-cadherin, and Pitx2 constituted a regulatory circuitry in the regulation of cell-cell interconnection. Furthermore, GSK3β-Ser21/9 interaction resulted in rephoshorylated NFAT1 in the nucleus (S4 Fig), preventing the binding of NFAT1 and DNA to consequently function for axonal growth as described above. These results suggested an integration of the TrkA/Akt2/GSK3β-Ser21/9/β-catenin and the RXRα/Gα_q/11_/CaN/NFAT1 pathways in the regulation of cell-cell interaction.

2) GSK3β-Tyr279/216 signaling: An active GSK3β-Tyr279/216 occurred within 8 hr, but not at 24 hr, resulting in the elevation of overall GSK3 protein (Fig 5A). It interacted to and activated native MAPT, exhibiting a prime effect via phosphorylation (Figs 4C and 5C). In turn, active MAPT bound to the microtubule component tubulins (Fig 5C), showing a co-localization immunocytochemically (Fig 5D) and also interacted with α-Synuclein by IP assay (Fig 5E), making MAPT as a central core regulator on the Parkin/MAPT/α-Synuclein/tubulins complexes. Co-localization of α-tubulin and Parkin was observed by imaging study (Fig 5D). To this end, we demonstrated that the TrkA/Akt2/GSK3β-Tyr279/216 cascades were involved in the initiation of MAPT function for microtubule assembly at the axonal growth cones. The apparent downregulation of GSK3β-Tyr279/216 after 8 hr (Fig 5A) probably protected neurites from degeneration compatible with the previous reports in Wallerian degeneration mice models [33]. Of particular clinical significance is that any abnormality of these biomarkers has been associated to the physiopathology of neurodegenerative disorder in association with PD and Alzheimer’s disease [34].

### 2. Regulation of *TH* Gene Expression at the Later Stage of Differentiation

**Fig 6:**
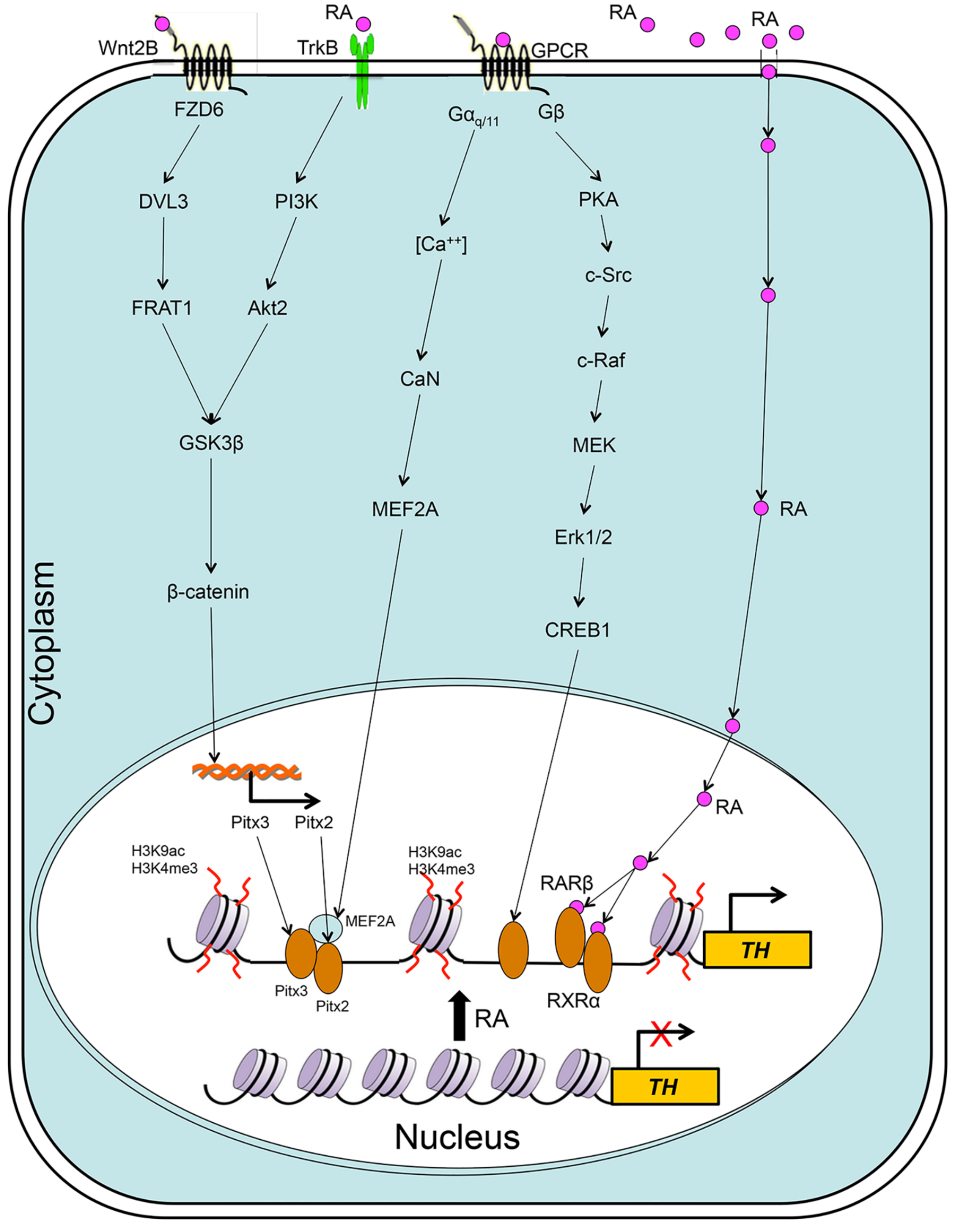
Schematic Illustration of a Variety of Distinct Transcriptional Pathways and Epigenetic Histone Modifications by RA Induction (24 hr) for *TH* Gene Expression in hTS Cells.

As differentiation proceeded to the later stage at 24 hr, cell susceptibility changed in response to RA, eliciting five distinct signaling pathways to coordinately target *TH* gene for transcription. Coincidentally, RA induced epigenetic histone modifications of H3K9ac and H3K4me3 at the DNA of *TH* gene. The orchestrated molecular events activated *TH* gene expression, generating DA committed progenitor cells as shown in a schematic illustration (Fig 6).

#### The genomic RA signaling pathway

**Fig 7:**
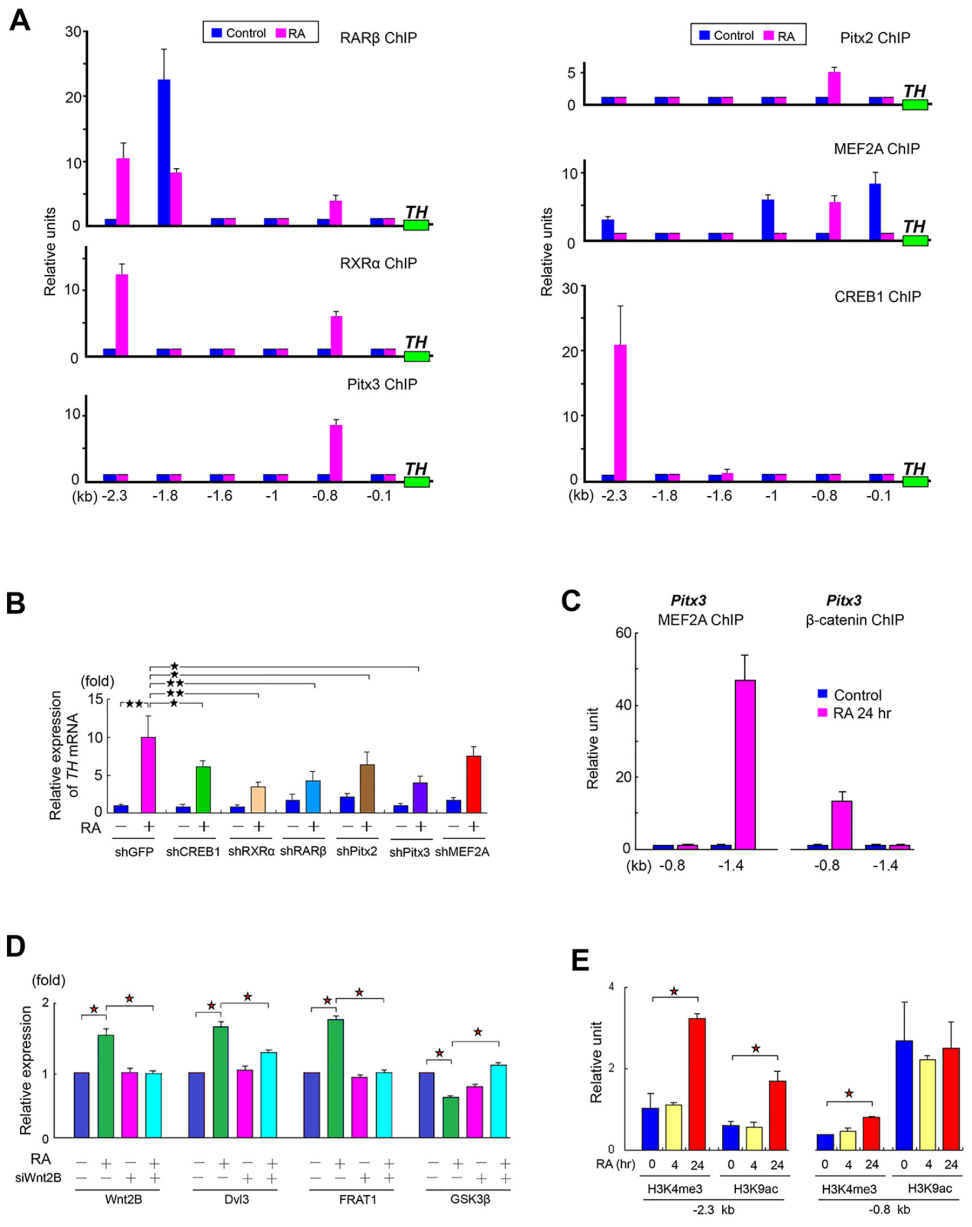
Activation of *TH* Gene Expression. (A) ChIP-qPCR analysis by RA induction (24 hr) showing: RARβ (upper), RXRα (middle) and Pitx3 (lower) at left panel as well as Pitx2 (upper), MEF2A (middle) and CREB1 (lower) at right panel to target the promoter of *TH* gene at different sites to produce the different *TH* levels in hTS cells. (n = 4) (B) qPCR analysis showing the relative *TH* mRNA expression and effects of knockdown of various transcription factors using specific shRNAs in hTS cells. (n = 4), shGFP as positive control. (C) ChIP-qPCR analysis showing the transcriptional ability of MEF2A (left) and β-catenin (right) targeting gene *Pitx3* at different sites. (n = 4) (D) Flow cytometric analysis of the effect of Wnt2B knockdown on the expressions of Wnt2B, Dvl3, FRAT1 and GSK3β at 24 hr of RA treatment. (n = 3) (E) RA activated H3K4me3 and H3K9ac at a different site of *TH* gene at 24 hr, but no change at 4 hr in hTS cells (n = 4). Data represent mean ± SD, ^★^: p < 0.05, ^★★^: p < 0.01.

Consistent with the well-known genomic RA signaling [6–7], we demonstrated that RA promoted the formation of RARβ/RXRα heterodimer by the nuclear cytoplasmic fractionation assay at 24 hr incubation (S5B Fig). This heterodimer then bound to the DNA-binding domain at two sites (-0.8 kb and -2.3 kb) of the regions known as RARE in *TH* gene by ChIP-qPCR analysis (Fig 7A). This action was further confirmed by using specific shRNAs against RARβ and RXRα that reduced *TH* mRNA expression (Fig 7B). We concluded that RA activated the genomic RA signaling pathway to promote *TH* gene transcription in hTS cells.

#### The TrkB/Akt2/GSK3β/β-Catenin/Pitx3 pathway

Notably, a desensitization of TrkA in response to RA occurred at 24 hr of induction (Fig 2A). Instead, TrkB was activated with consequent downstream Akt2, but not Akt1 and Akt3 (Fig 2A and 2B). Knockdown of TrkB reduced Akt2 expression, thereby, establishing the TrkB/PI3K/Akt2 pathway. Subsequently, Akt2 resulted in the phosphorylation of GSK3β at Ser21/9 site (Fig 5A), resulting in the activation and nuclear translocation of β-catenin (S5B Fig). In the nucleus, β-catenin targeted at the gene *Pitx3* to produce Pitx3 (Fig 7C). In turn, Pitx3 enabled transcription of gene *TH* (Fig 7A). Knockdown of Pitx3 reduced the *TH* mRNA expression as well (Fig 7B). We concluded that RA induced the activation of TrkB/PI3K/Akt2/GSK3β-Ser21/9/β-catenin signaling pathway to promote *TH* gene transcription mediating Pitx3.

#### The canonical Wnt2B/Fzd6/β-catenin/Pitx2 pathway

Wnt/β-catenin pathway is well-known for its pivotal impact on PD disease [35]. Among them, Wnt2 is initially expressed at the midbrain in mice that regulates progenitor proliferation in the developing brain [36]. Based on our previous DNA microarray analysis [5], we constructed a predictive canonical Wnt2B/Fzd6/Dvl3/FRAT1/GSK3β-Ser21/9 signaling pathway by a series of expressions in its related mRNAs (S6A Fig). For identification, flow cytometry revealed an upregulation of Wnt2B with its downstream effectors, including mediator protein Dishevelled 3 (Dvl3), proto-oncogene FRAT1 and a downregulated GSK3β-Ser21/9 (Fig 7D). Knockdown of Wnt2B reduced expressions of Wnt2B, Dvl3, and FRAT1, but increased GSK3β-Ser21/9 activity (Fig 7D and 7B). Subsequently, the inhibitory GSK3β-Ser21/9 led to the nuclear translocation of β-catenin. In the nucleus, β-catenin induced transcription of genes *Pitx3* (Fig 7A) and *Pitx2* (Fig 4I and Fig 7A). In turn, both of Pitx3 and Pitx2 were able to target at the similar promoter of gene *TH* for TH expression. Knockdown of both Pitx3 and Pitx2 reduced expression of *TH* mRNA (Fig 7B). These results demonstrated that RA induced the canonical Wnt2B/Fzd6/β-catenin signaling pathway to produce Pitx3 and Pitx2, which in turn targeted at gene *TH* for transcription.

#### The Gα_q/11_/CaN/MEF2A pathway

Unlike transient CaMKII, CaN activities were able to sustain up to 24 hr during the differentiation (Fig 4D), suggesting a capacity in continuous dephosphorylation of MEF2A for biological function. A nuclear cytoplasmic fractionation analysis revealed the nuclear entrance of MEF2A at 24 hr induction, which action was supported by using CaN inhibitor cyclosporine (CsA) (S6B Fig). In the nucleus, MEF2A targeted at gene *TH* (Fig 7A). Nevertheless, knockdown of MEF2A did not show reduction of *TH* mRNA expression (Fig 7B). These results implicated that though MEF2A was not a direct transcription factor for *TH* gene, MEF2A was a co-activator of Pitx2 for *TH* gene transcription.

#### The Gβ/PKA/MEK/Erk1/2/CREB1 pathway

Given a linkage between GPCRs and mitogen-activated protein kinases (MAPK) by multiple pathways described previously [37], we demonstrated that the RA-activated Gβ promoted the expression of protein kinase A (PKA) at 24 hr induction. This consequently activated a series of protein-protein interactions with adaptor molecules, including proto-oncogene proteins c-Src, c-Raf, MEK, and Erk1/2 to establish the PKA/c-Src/c-Raf/p-MEK/p-Erk1/2 pathway (Fig 4A and 4B). This pathway was further supported by the inhibitory action of Src-selective kinase inhibitor PP1 (Fig 4A *)*, Gβ shRNAs (Fig 4B), and MAPK/Erk kinase inhibitor PD98059 (S1G Fig). Subsequently, Erk1/2 activation resulted in the phosphorylation of CREB1, leading to the nuclear translocation of CREB1. In the nucleus, CREB1 targeted to the *TH* promoter at -2.3 kb site, but not -0.8 kb site, to activate *TH* transcription (Fig 7A) to produce TH (S6C Fig). Knockdown of CREB1 reduced the expression of *TH* mRNA by qPCR and immunoblotting assays (Fig 7B and S6D Fig). Together, we demonstrated that RA induced activation of the Gβ/PKA/Erk1/2/CREB1 pathway for *TH* gene transcription. To this end, we concluded that RA induced DA committed progenitor cells in hTS cells via coordination of Wnt2B/Fzd6, TrkB/Akt2, G proteins, and genomic RA signaling pathways.

#### Epigenetic histone modifications of H3K4me3 and H3K9ac

Epigenetic histone modifications play critical roles on chromatin remodeling and provide insights to further understand the molecular mechanisms in genetic regulation. Much foci have been on the effects of tail acetylation of histone H3 (H3K9ac) and trimethylation of H3 lysine 4 (H3K4me3) by ChIP-qPCR analysis in *TH* gene due to enrichments in the promoter regions and the elevated levels surrounding transcription sites (TSS) of active genes by large-scale and genome-wide analyses [38–39].

Constitutively, we found that H3K4me3 expressed a relatively silent level at both -0.8 kb and -2.3 kb sites of the promoter of *TH*, but H3K9ac expressed a relatively higher level (>2-fold) at -0.8 kb site than -2.3 kb site in hTS cells (Fig 6E). At 4 hr induction, RA did not change both H3K4me3 and H3K9ac levels, suggesting a condensed chromatin status. This may explain why RA did not activate *TH* gene at the early differentiation. However, H3K4me3 significantly elevated at both -0.8 kb and -2.3 kb sites at 24 hr induction; while H3K9ac elevated significantly only at -2.3 kb site (Fig 6E). Consequently, the chromatins of *TH* gene relaxed, which facilitated the binding of transcription factors to DNA. Taken all together, we concluded that the epigenetic histone modifications (i.e., H3K4me3 and H3K9ac) and the *TH* gene transcription factors (i.e., Pitx2, Pitx3, MEF2A, CREB1, RAR and RXR) orchestrated the differentiation of DA committed progenitor cells, exhibiting stage-specific and cell-type dependent characteristics in hTS cells.

#### Postimplantation co-expression of TH and CREB1 in the lesioned SNC of PD rat model

**Fig 8:**
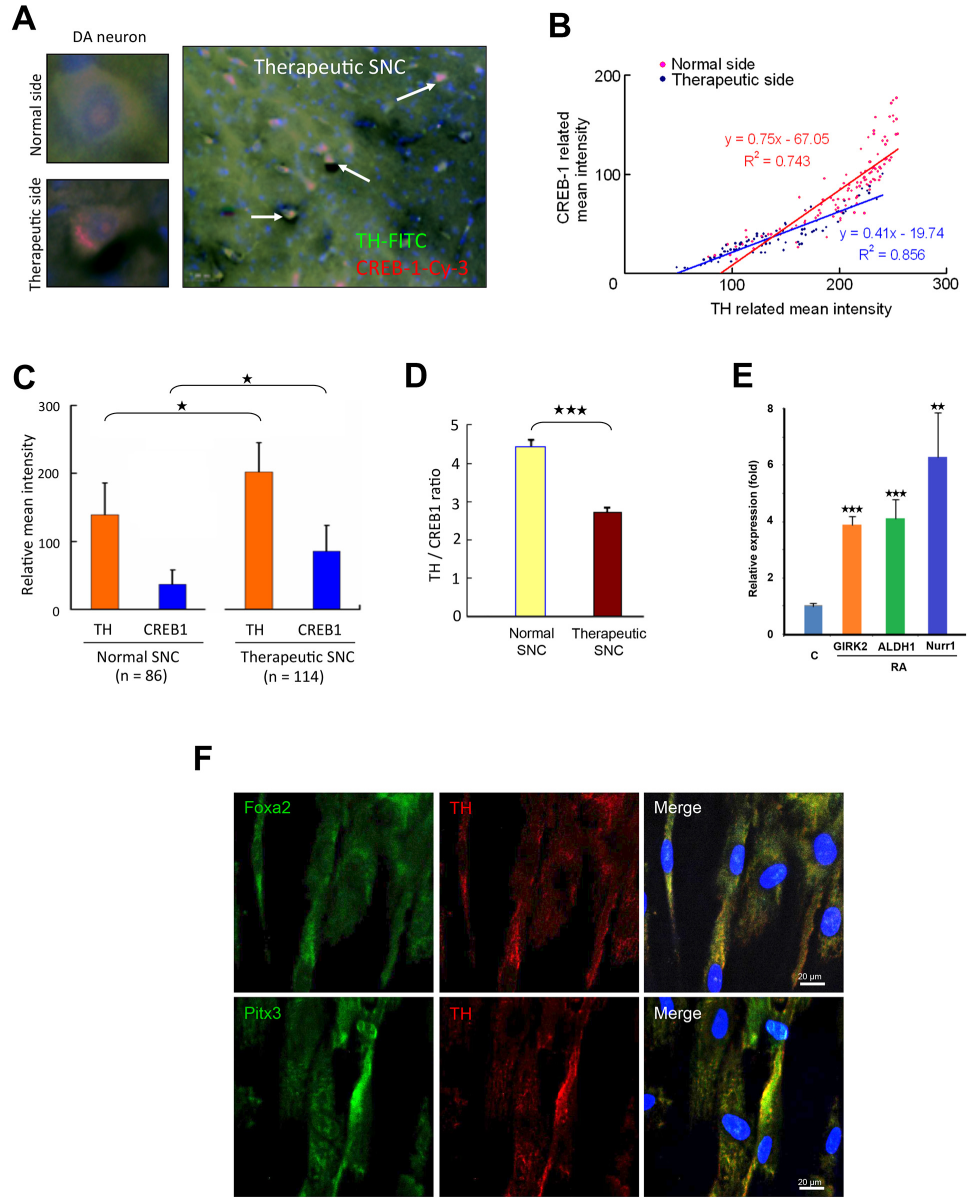
TissueQuest Analysis of TH and CREB1 Expressions *in vivo*. (A) Left panel: Expression of immunoreactive CREB1 (red) and TH (green) in DA neurons in normal SNC (upper) and therapeutic SNC (lower). Right panel: Colocalization of TH and CREB1 in the regenerated DA neurons (arrow) of therapeutic SNC at 12-week postimplantation of the hTS cell-derived NSCs in PD rat brain. (B) A distributive correlation of CREB1 and TH intensity in DA neurons counted from the normal SNC and the therapeutic SNC after 12-week implantation. R^2^ = 0.856. (C-D) Comparison of relative expression of TH and CREB1 between the normal DA neurons (left, n = 86 cells counted) and the regenerated DA neurons (right, n = 114 cells counted) in a brain section at postimplantation (C) and the mean intensity of TH to CREB1 ratio in DA neurons is significantly lower in therapeutic SNC than normal SNC (D). (E) RA increases levels of GIRK2, ALDH1, and Nurr1 mRNAs after one-day incubation by qPCR assay. (F) Immunofluorescence imaging reveals co-expression of TH with Foxa2 and Pitx3. n = 4. Data represent mean ± SD, ^★^: p < 0.05, ^★★^: p<0.01, ^★★★^: p < 0.001.

To validate the expression of RA-induced TH *in vivo*, we examined the 6-OHDA-lesioned substantia nigra compacta (SNC) of PD rats that received intracranial implantation of the hTS cell-derived NSCs generated by RA [5]. By immunocytochemistry, we found co-expressions of CREB1 and TH in the DA neurons of both therapeutic SNC and normal SNC at 12 weeks postimplantation (Fig 8A). Quantitative analysis revealed a correlative intensity of immunoreactive CREB1 and TH in both therapeutic and normal side (Fig 8B). Both CREB1 and TH intensities were higher in the newly generated DA neurons of the therapeutic SNC than the normal side (Fig 8C). The ratio of TH to CREB1, however, was lower in the therapeutic side than the normal side (Fig 8D). These results suggested that the *in vitro* expressions of CREB1 and TH were similar to that of the *in vivo* regenerated DA neurons after implantation. This suggests that hTS cell-derived NSCs can continuously differentiate and contain the capacity to generate DA neurons in the lesioned SNC. To further characterize the DA committed progenitor cells, we demonstrated the transcriptional expressions of functional A9 midbrain dopaminergic neurons in the substantia nigra and ventral tegmental areas [40–43]. We showed expressions of GIRK2, ALDH1, and Nurr1 mRNAs by qPCR assays (Fig 8E) and the co-expression of Pitx3 and TH as well as Foxa2 and TH by immunofluoresence imaging studies (Fig 8F).

## Discussion

Transition of pluripotent stem cells to NSCs requires two cellular processes: i) morphologic changes via axonogenesis and ii) NSC-associated gene expression, by which NSCs integrate into the microenvironments of the brain after implantation to generate specific types of neural cells, ultimately developing functional neuronal connections. Several signaling pathways are involved in the regulation of neural stem cell transition, including fibroblast growth factor (FGF) pathway [44], Notch pathway [45], sonic hedgehog (SHH) pathway [46], Wnt pathway [47], BMP [48], and NFκB pathway [49]. Here, we report a robust molecular network of RA induction in the differentiation of hTS cells to DA committed progenitor cells, exhibiting spatiotemporal and stage-specific characteristics. At the early stage, RA induced the interplay of TrkA and G protein signaling pathways to neural fate by activating molecules in association with microtubule assembly and stabilization, axonal growth, and morphogenesis. At the later stage, in addition to TrkB and G protein signaling, RA further induced the Wnt2B and genomic signaling pathways, along with epigenetic histone modifications for *TH* gene specification.

RA activates many receptors including TrkA and TrkB via neurotrophin/Trk signaling to promote differentiation of neuronal precursors [50]. In hTS cells, RA activated TrkA/Akt3/mTOR pathway to trigger subcellular localization of RARβ and RXRα, which immediately recruited Gβ and Gα_q/11_ to form the RARβ/Gβ and RXRα/Gα_q/11_ complexes, respectively. This highlights a novel functional link between Trk nuclear receptors and G proteins. These complexes initiated novel non-genomic RA signaling pathways, which may feasibly support the association of RARβ and RXRα with growth cone guidance for chemoattraction by eliciting turning in neurite outgrowth [51] and the fast formation of RARα/Gαq complexes in lipid rafts as a requirement for the activation of p38MAPK in response to RA [46]. The interaction of RXRα and Gα_q/11_ is most likely attributed to human Gα_q/11_ containing a single LXXLL motif (where L is leucine; and X is any amino acid) in the N-terminal region, which is required for its ligand-dependent interaction with nuclear receptors [52–53]. The interaction of RARβ and Gβ remains unclear.

Local translation of mRNA in axon guidance has been challenging, partly because of the technical barrier of inhibiting protein synthesis exclusively in the axonal compartment [54]. NGF/TrkA signaling induces local translation of CREB mRNA at axonal growth cones [55]. In hTS cells, the synchronization of TrkA/PI3K/Akt1 and the RXRα/Gα_q/11_/CaMKII pathways enhanced CREB1 signal strength by the auto-phosphorylation of CaMKII at the cell surface [24]. Consequently, CREB1 activated *MAPT* gene transcription in the nucleus, leading to the transport of *MAPT* transcripts towards the growth cones and/or along the axon, where local translation may occur [56]. However, functions of MAPT regulated a site-specific phosphorylation event where phosphorylation of GSK3β-Tyr279/216 primed the native MAPT to initiate the binding to MAPT and tubulins in the microtubules assembly. This suggests that GSK3β regulates the microtubule-binding domain on MAPT molecule, making MAPT a core regulator in growth cones under normal physiological conditions. Furthermore, the RXRα/Gα_q/11_/CaN signaling resulted in the dephosphorylation of MEF2A for nuclear translocation to activate the transcriptional expression of α-Synuclein and Parkin. As a result, the regulatory MAPT-centered protein complexes were formed, including MAPT, α-Synuclein, Parkin, α- and β-tubulins, for microtubule organization and stabilization during axonal growth.

In neurons, intracellular calcium levels are crucial guidance cues in the growth cones, depending on the stage-specific balance among the ER, VGCCs, and CRACs activities [57]. Intracellular calcium levels determine the distribution of its responsive effectors CaMKII and CaN. Since attraction primarily is mediated by CaMKII and repulsion by CaN, the axon will grow towards the side of the compartment with a greater CaMKII to CaN ratio [58]. In hTS cells, RXRα/Gα_q/11_ signaling initially induced a higher CaMKII/CaN ratio at 30 min, but this ratio downregulated after 4 hr (Fig 4D). These temporal changes in the CaMKII/CaN ratios during neurodifferentiation may guide axonal movements to the appropriate spatial destinations in a developing brain.

In addition, the linkage of TrkA/PI3K/Akt signaling and axonal characteristics is based on the experiments by William D. Snider’s lab [59–60]. For example, both PI3K/Akt and Ras/Raf/MEK pathways activate NGF-induced axon elongation, wherein PI3K increases axon calibers and cell bodies with little effect on axon length while Akt causes axon thickening and distal branching, but not significant elongation. Moreover, Raf-1 produces extensive growth of relatively thin axonal processes and the enlarged cell body. Interestingly, PI3K/Akt signaling pathway also controls the synaptogenesis and spinogenesis in hippocampal neurons [61]. In hTS cells, we found that TrkA/Akt2/GSK3β signaling regulated cell adhesions via activation of subcellular β-catenin and/or β-catenin/N-cadherin complex at the cell border. This suggests that TrkA and G protein signaling were involved in the activation of effector genes, towards a neural morphology.

As differentiation progressed, cellular susceptibility to RA switched from TrkA to TrkB at the cell membrane. This is compatible with reports that RA suppresses TrkA mRNA but induces TrkB mRNA expression in the sympathetic neurons [62]; thereby, no trophic action with NGF/TrkA on DA neurons is observed in SNC, but TrkB signaling is associated with dopamine release in the early mesencephalic neurons [63]. The mechanism of how such desensitization occurs is not understood. However, desensitization of receptor signaling may determine physiological fate during differentiation.

Notably, both Pitx2 and Pitx3 were required for the *TH* gene expression in hTS cell-derived DA committed progenitor cells. However, very little or no evidence has been reported for the presence of Pitx2 in SNC. Pitx2 is required for the midbrain development and loss of Pitx2 disrupts differentiation of subthalamic nucleus neurons and axonal outgrowth [64]. While functionally associated with GABA synthesis, Pitx2 is also necessary for tract formation for both DA and GABA neuron migration in the mesencephalon of a developing brain [65]. Interestingly, we found that MEF2A is an essential co-activator of Pitx2 in activating *TH* gene expression. Furthermore, Pitx3 is selectively expressed in the mesencephalic dopaminergic SNC for the survival of DA neurons [66]. In mice, the absence of Pitx3 impairs the DA neuronal migration in the SNC [67]. A further study to compare the interaction of Pitx2 and Pitx3 on DA and GABA neurodifferentiation is suggested to clarify this observation.

Histone modification is associated with transcriptionally repressive or active conformation of gene expression. Methylation of lysine at the fourth residue of histone 3 (H3K4me) promotes active conformation, but at the ninth residue (H3K9me) promotes repressive conformation; thereby, affecting the chromatin by recruiting protein complexes to regulate transcription [68]. In the chromatin of TH-positive cells, a correlation exists between TH activity and H3 and H4 expressions [69]. Relevantly, H3K4me3 has been expressed in the undifferentiated progenitors within the subventricular zone of the brain, where postnatal neurogenesis occurs [70]. In hTS cells, RA increased levels of both H3K4me3 and H3K9ac, which consequently contributed to chromatin relaxation that facilitated *TH* gene activation at 24 hr induction. However, no such histone modifications were observed at the early 4 hr induction, suggesting in a condensed chromatin structure that prevented the binding of Pitx2, Pitx3, MEF2A, and CREB1 to *TH* gene. These active molecules may serve for axonal assembly and growth in morphological changes. Ultimately, an evaluation of both the genetic transcriptions and the epigenetic modifications may provide a more accurate prediction in the gene regulation for biological functions. Nevertheless, despite what regulatory mechanisms have been achieved in the understanding of differentiation of pluripotent stem cells to NSCs *in vitro*, without further advancements in technology, clinical limitations will exist because we cannot observe cellular behavior and fate after implantation *in vivo*.

## Supporting Information

S1 Fig Downstream effectors of RA-induced TrkA signaling. (A) RA (10 μM, 4 hr) activated all Akt subunits and these actions were inhibited by Wortmannin (100 nM) by flow cytometry. (B) Only Akt3, not Akt1 and Akt2, interacted with mTOR by IP. (C) RA enhanced the interaction of Akt3 and mTOR (Ser2448) by IP assay. (D) Knockdown of Akt3 reduced the expression of mTOR. Scramble: positive control, β-actin: loading control. (E) RA enhanced the interaction of mTOR and 4EBP1 by IP assay. (F) RA induced phosphorylation of CREB1 was inhibited by Akt inhibitor MK2206, MAPK/Erk kinase inhibitor PD98059, and CaMKII inhibitor KN93 at 4 hr induction. C as control. (G) RA induced phosphorylation of c-Raf, Mek, Erk1/2, and CREB1 by immunoblotting assay. MAPK/Erk kinase inhibitor PD98059 decreased the phosphorylation of Erk1/2 and CREB1 at both 4 and 24 hr incubation. β-actin as loading control.
(TIF)

S2 Fig RARβ/Gβ/ER calcium signaling and its downstream effectors. (A) RA increased intracellular calcium levels (n = 13 cells counted), which was inhibited by pre-incubation of IP3 receptor antagonist 2-APB in a dose-dependent manner (R2 = 0.8984; n = 10) analyzed by real-time cell imaging microscopy. (B) RA (4 hr) induced interaction of CaMKII and CREB1 by IP assay. C as control. IgG as negative control. (C) RA (4 hr) promoted expressions of CaMKII, CaN, NFAT1 and MEF2A, which were inhibited by (50 μM, 2 hr). β-actin as loading control.
(TIF)

S3 Fig Regulation of intracellular calcium levels. (A-B) IP assays showed that RA (10 μM, 4 hr) enhanced the interaction of MEF2A and NFAT1, (A) and interaction between CaMKII and Parkin as well as Parkin and MAPT (B). C as control. IgG as negative control. (C) RA (10 μM) induced [Ca^++^] signal in hTS cells cultivated in calcium free medium. After total calcium releases, the intracellular calcium levels elevated by adding extrinsic CaCl_2_ (2 mM, n = 124 cells counted, 30 min; upper panel) and by adding extrinsic KCl (60 mM; n = 45 cells counted; 30 min; middle panel). The KCl-induced calcium elevation was attenuated by pretreatment of nifedipine (5 μM; n = 72 cells counted; 30 min; lower panel). Cells were stained with a Ca^2+^-specific dye Fura-2 in HBSS buffer for 20 min at room temperature to measure the calcium responses by real-time cell imaging microscopy (Olympus, Cell-R) and Olympus Cell-R imaging software. The Ca^2+^-free medium contains EGTA (1.2 mM/L, Applichem) and Thapsigargin (10 mM/L, Calbiochem).
(TIF)

S4 Fig Effects of GSK3β on the CaN/NFAT1 pathway. (A) RA (10 μM, 4 hr) enhanced the interaction of importin and NFAT1 by IP assay. C as control. IgG as negative control. (B) RA increased nuclear translocalization of NFAT1 by nucleocytoplasmic fractionation assay. Lamin A/C as marker of the nuclear extract. α-tubulin as marker of the cytoplasmic extract. (C-D) RA (10 μM, 4 hr) enhanced the interaction of GSK3β -Ser21/6) and NFAT1, (C) as well as MEF2A interacted to both NFAT1 and Pitx2 bi IP assay (D). (E) Knockdown of NFAT1 reduced the expression of MEF2A by immunoblotting assay. Scramble as positive control. β-actin as loading control.
(TIF)

S5 Fig Activation of Akt2/GSK3β/β-catenin/N-cadherin pathway. (A) RA (10 μM, 4 hr) enhanced the interaction of Akt2 and GSK3β. C as control. IgG as negative control. (B) RA induced nuclear translocalization of β-catenin by nucleocytoplasmic fractionation assay. Lamin A/C as marker of the nuclear extract. α-tubulin as marker of the cytoplasmic extract. C as control. (C) RA (10 μM, 4 hr) enhanced the interaction of β-catenin and LEF1 by IP assay. C as control. IgG as negative control.
(TIF)

S6 Fig Activation of TH expression. (A) Relative expressions of components in the Wnt2B/Fzd6/Dvdl3/FRAT1/GSK3β signaling pathway assessed by DNA microarray assessment in hTS cell after treatment with RA (10 μM) for 24 hr. (B) Nuclear cytoplasmic fractionation analysis revealed the entrance of MEF2A into nucleus at 24 hr of RA induction, which was inhibited by CaN inhibitor cyclosporine (CsA, 5 μM). (C-D) RA induced expressions of CREB1, Pitx2 and TH at 24 hr induction by immunoblotting assay (C) and this action was inhibited by using siRNA against CREB1 (D). Scramble as positive control, β-actin as loading control.
(TIF)

S1 Table Reagents used in this study. (DOCX)

S2 Table Primary and second antibodies used in this study. (DOCX)

S3 Table siRNAs used in this study. (DOCX)

S4 Table Primers used in this study. (DOCX)

S1 Text Supporting Information Text. (DOCX)
