# Supplementary figures and images for: Dynamic Trk and G Protein Signalings Regulate Dopaminergic Neurodifferentiation in Human Trophoblast Stem Cells
Source: PLoS One. 2015 Nov 25;10(11):e0143852. doi: 10.1371/journal.pone.0143852 (PMC4659658; doi:10.1371/journal.pone.0143852)

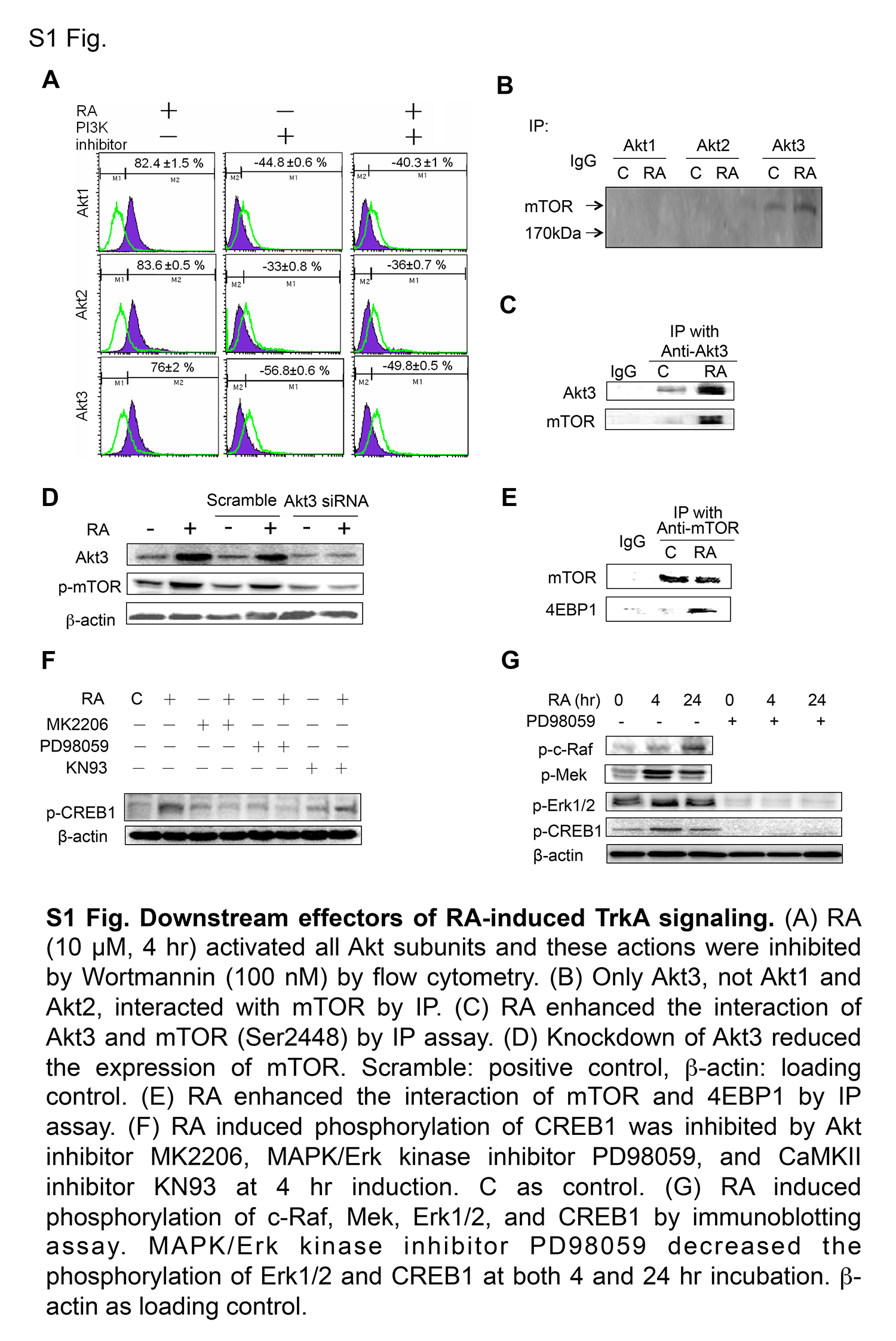

Supplement: S1 Fig — (A) RA (10 μM, 4 hr) activated all Akt subunits and these actions were inhibited by Wortmannin (100 nM) by flow cytometry. (B) Only Akt3, not Akt1 and Akt2, interacted with mTOR by IP. (C) RA enhanced the interaction of Akt3 and mTOR (Ser2448) by IP assay. (D) Knockdown of Akt3 reduced the expression of mTOR. Scramble: positive control, β-actin: loading control. (E) RA enhanced the interaction of mTOR and 4EBP1 by IP assay. (F) RA induced phosphorylation of CREB1 was inhibited by Akt inhibitor MK2206, MAPK/Erk kinase inhibitor PD98059, and CaMKII inhibitor KN93 at 4 hr induction. C as control. (G) RA induced phosphorylation of c-Raf, Mek, Erk1/2, and CREB1 by immunoblotting assay. MAPK/Erk kinase inhibitor PD98059 decreased the phosphorylation of Erk1/2 and CREB1 at both 4 and 24 hr incubation. β-actin as loading control. (TIF) [file pone.0143852.s001.tif]

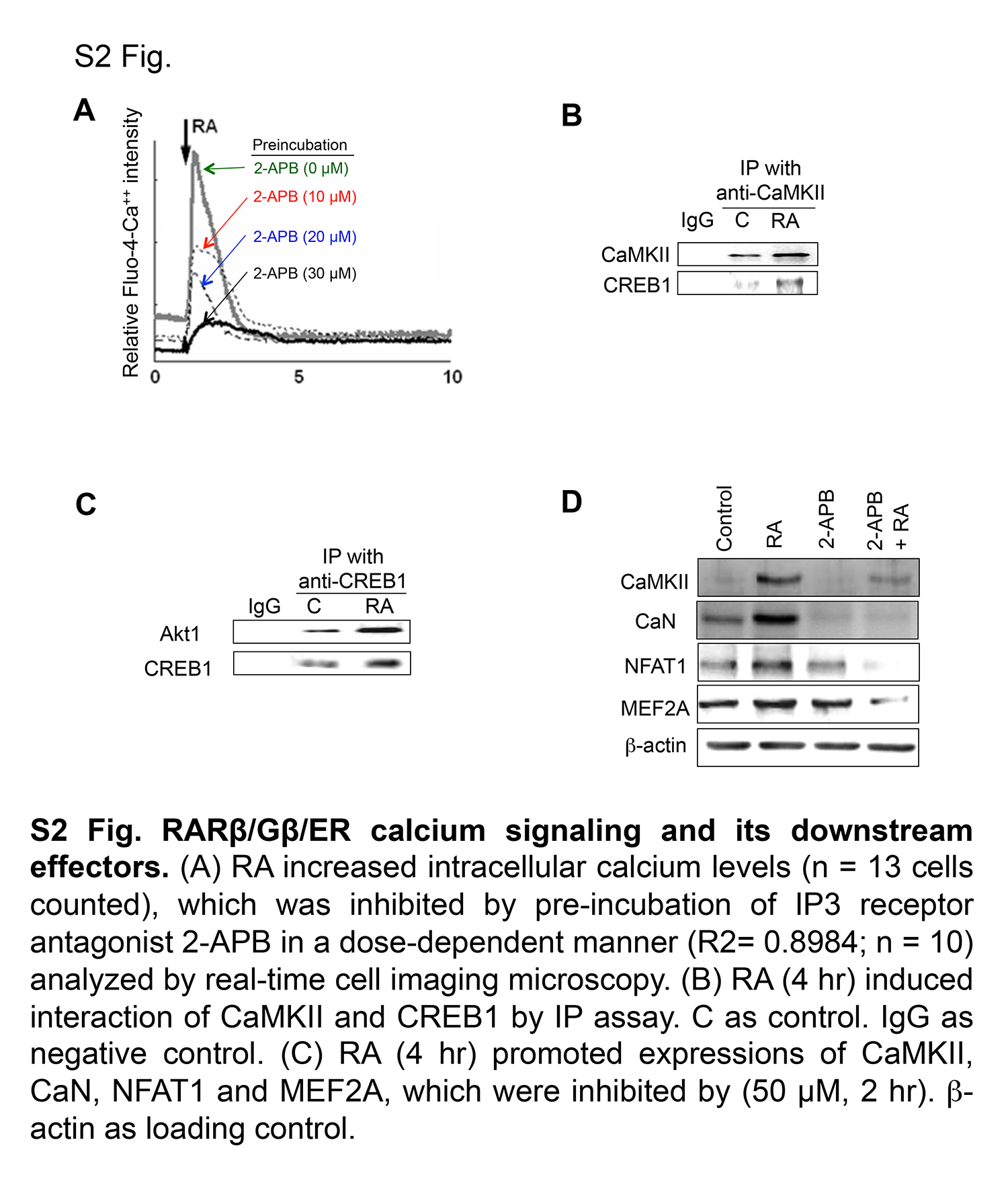

Supplement: S2 Fig — (A) RA increased intracellular calcium levels (n = 13 cells counted), which was inhibited by pre-incubation of IP3 receptor antagonist 2-APB in a dose-dependent manner (R2 = 0.8984; n = 10) analyzed by real-time cell imaging microscopy. (B) RA (4 hr) induced interaction of CaMKII and CREB1 by IP assay. C as control. IgG as negative control. (C) RA (4 hr) promoted expressions of CaMKII, CaN, NFAT1 and MEF2A, which were inhibited by (50 μM, 2 hr). β-actin as loading control. (TIF) [file pone.0143852.s002.tif]

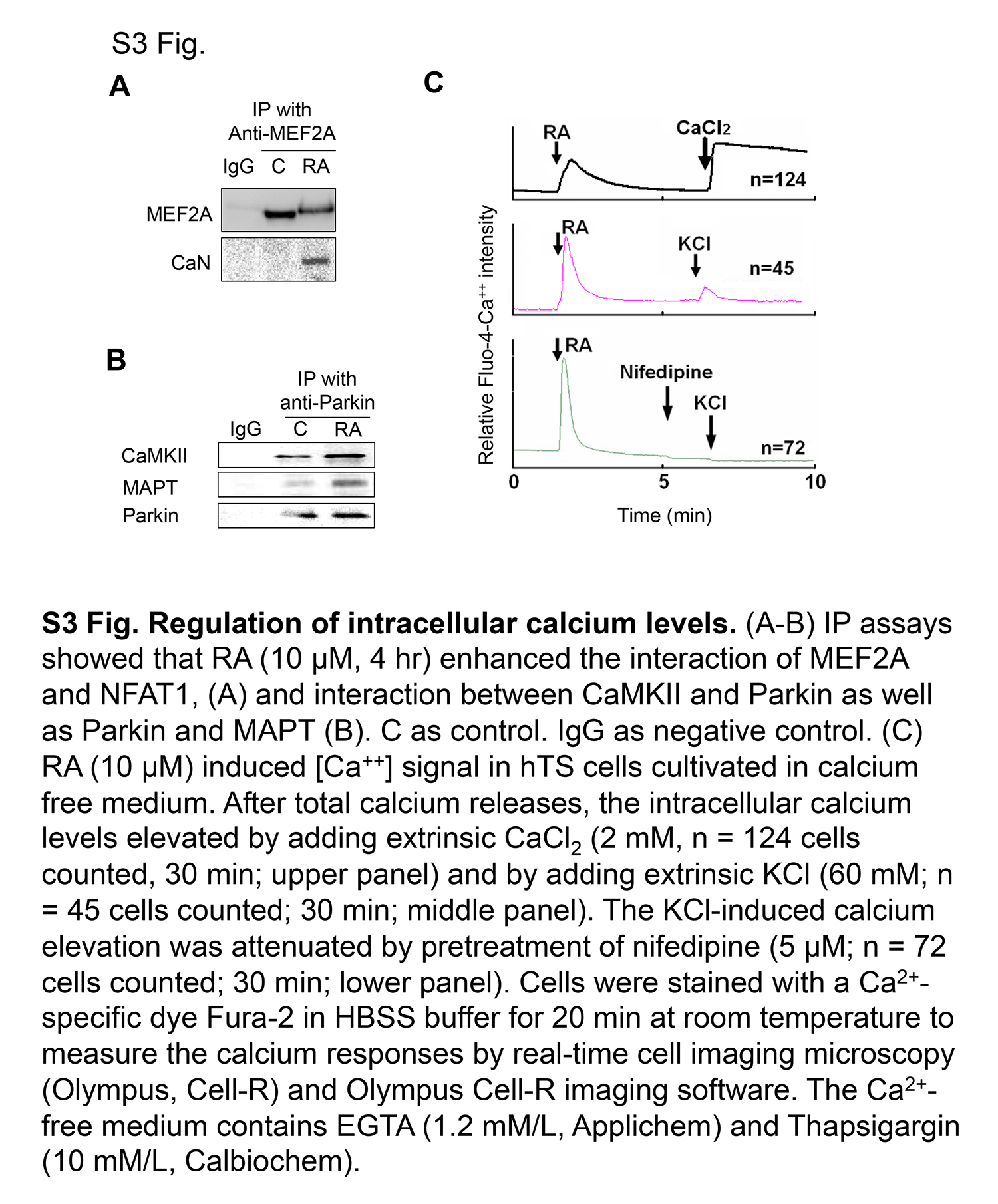

Supplement: S3 Fig — (A-B) IP assays showed that RA (10 μM, 4 hr) enhanced the interaction of MEF2A and NFAT1, (A) and interaction between CaMKII and Parkin as well as Parkin and MAPT (B). C as control. IgG as negative control. (C) RA (10 μM) induced [Ca++] signal in hTS cells cultivated in calcium free medium. After total calcium releases, the intracellular calcium levels elevated by adding extrinsic CaCl2 (2 mM, n = 124 cells counted, 30 min; upper panel) and by adding extrinsic KCl (60 mM; n = 45 cells counted; 30 min; middle panel). The KCl-induced calcium elevation was attenuated by pretreatment of nifedipine (5 μM; n = 72 cells counted; 30 min; lower panel). Cells were stained with a Ca2+-specific dye Fura-2 in HBSS buffer for 20 min at room temperature to measure the calcium responses by real-time cell imaging microscopy (Olympus, Cell-R) and Olympus Cell-R imaging software. The Ca2+-free medium contains EGTA (1.2 mM/L, Applichem) and Thapsigargin (10 mM/L, Calbiochem). (TIF) [file pone.0143852.s003.tif]

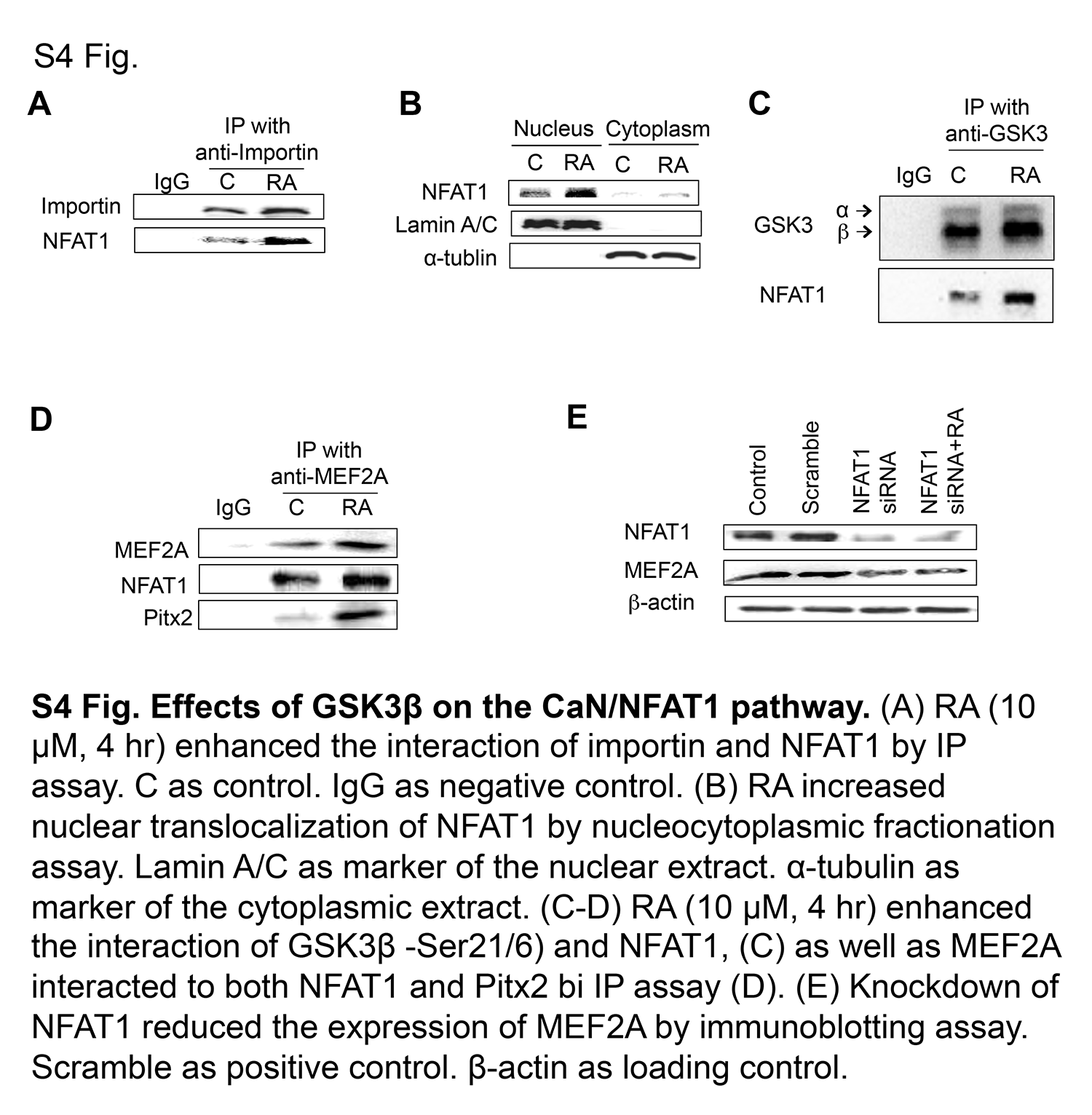

Supplement: S4 Fig — (A) RA (10 μM, 4 hr) enhanced the interaction of importin and NFAT1 by IP assay. C as control. IgG as negative control. (B) RA increased nuclear translocalization of NFAT1 by nucleocytoplasmic fractionation assay. Lamin A/C as marker of the nuclear extract. α-tubulin as marker of the cytoplasmic extract. (C-D) RA (10 μM, 4 hr) enhanced the interaction of GSK3β -Ser21/6) and NFAT1, (C) as well as MEF2A interacted to both NFAT1 and Pitx2 bi IP assay (D). (E) Knockdown of NFAT1 reduced the expression of MEF2A by immunoblotting assay. Scramble as positive control. β-actin as loading control. (TIF) [file pone.0143852.s004.tif]

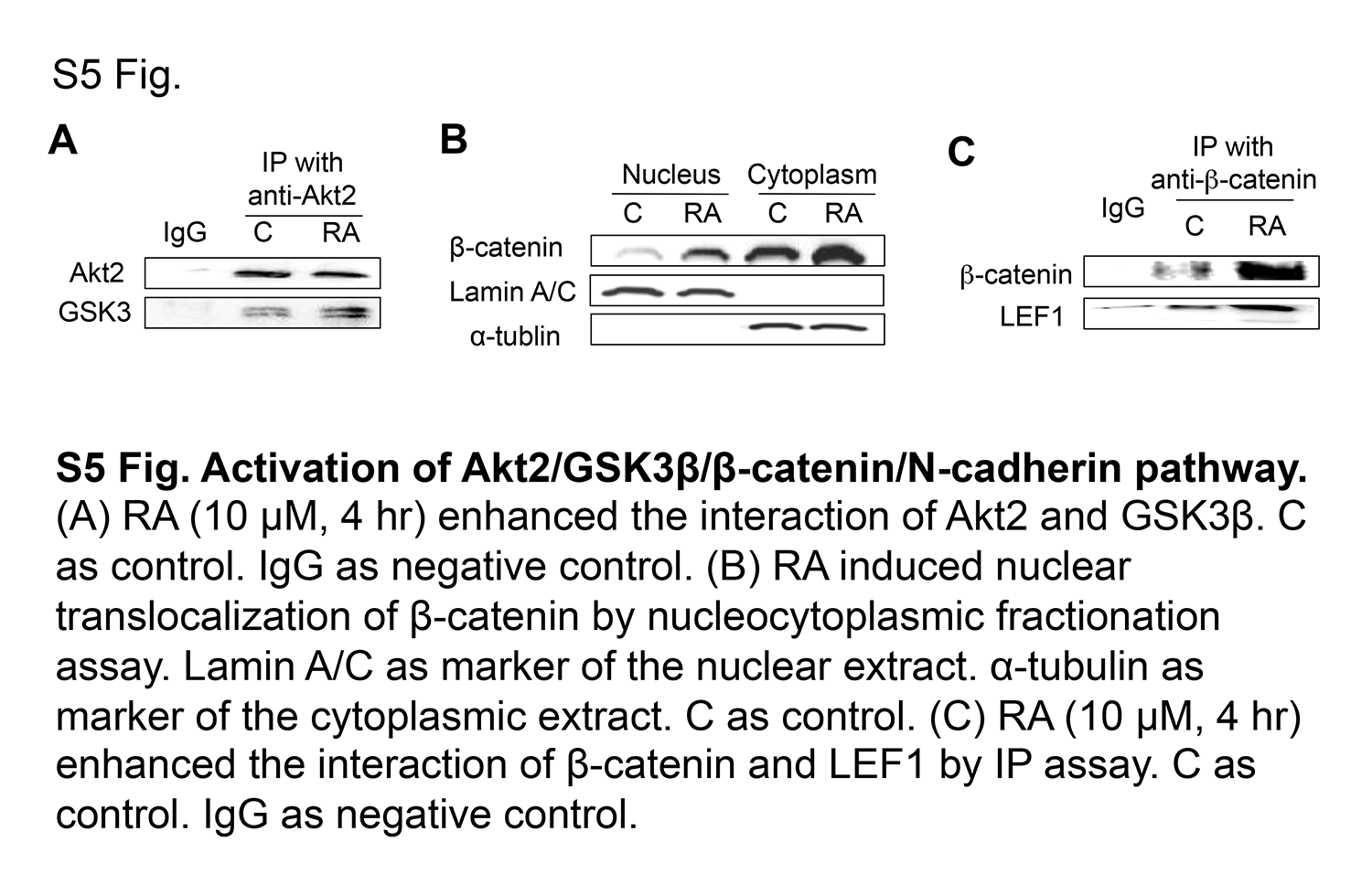

Supplement: S5 Fig — (A) RA (10 μM, 4 hr) enhanced the interaction of Akt2 and GSK3β. C as control. IgG as negative control. (B) RA induced nuclear translocalization of β-catenin by nucleocytoplasmic fractionation assay. Lamin A/C as marker of the nuclear extract. α-tubulin as marker of the cytoplasmic extract. C as control. (C) RA (10 μM, 4 hr) enhanced the interaction of β-catenin and LEF1 by IP assay. C as control. IgG as negative control. (TIF) [file pone.0143852.s005.tif]

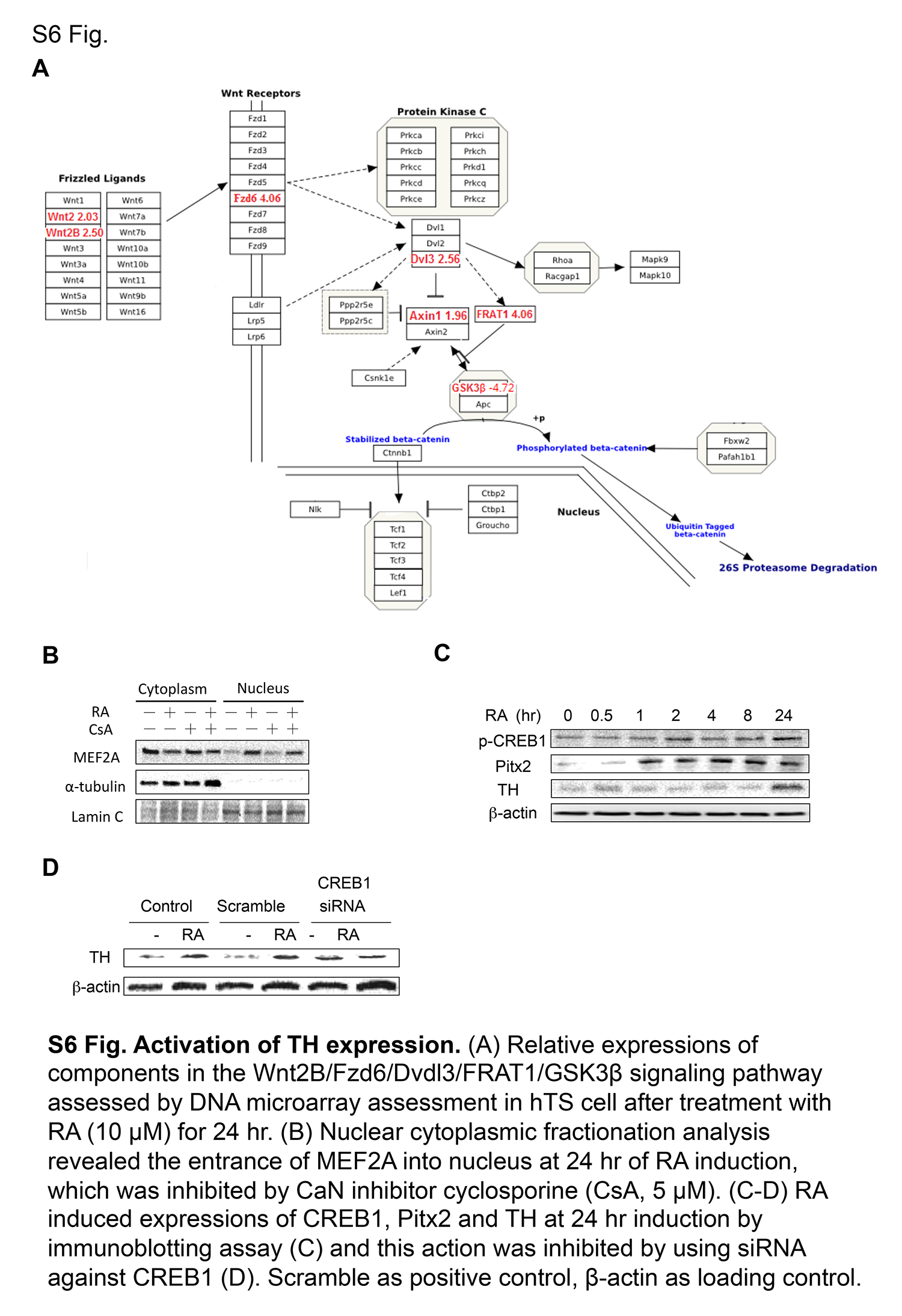

Supplement: S6 Fig — (A) Relative expressions of components in the Wnt2B/Fzd6/Dvdl3/FRAT1/GSK3β signaling pathway assessed by DNA microarray assessment in hTS cell after treatment with RA (10 μM) for 24 hr. (B) Nuclear cytoplasmic fractionation analysis revealed the entrance of MEF2A into nucleus at 24 hr of RA induction, which was inhibited by CaN inhibitor cyclosporine (CsA, 5 μM). (C-D) RA induced expressions of CREB1, Pitx2 and TH at 24 hr induction by immunoblotting assay (C) and this action was inhibited by using siRNA against CREB1 (D). Scramble as positive control, β-actin as loading control. (TIF) [file pone.0143852.s006.tif]
