# Supplementary material for: Dynamic Trk and G Protein Signalings Regulate Dopaminergic Neurodifferentiation in Human Trophoblast Stem Cells
Source: PLoS One. 2015 Nov 25;10(11):e0143852. doi: 10.1371/journal.pone.0143852 (PMC4659658; doi:10.1371/journal.pone.0143852)
Supplement: S1 Table — (DOCX) [file pone.0143852.s007.docx]

**S1 Table. Reagents used in this study**

| Reagent | Product | Concentration | Manufacturer |
| --- | --- | --- | --- |
| Retinoic acid |  | 10 μM | Sigma |
| GNF-5837 |  | 18 nM | Millipore |
| Cycloheximide |  | 100 μg/ml | Sigma |
| Actinomycin D |  | 10 μg/ml | Sigma |
| Wortmannin |  | 100 nM | Calciochem |
| 4',6-diamidino-2-phenylindole |  | 10 µg/ml | Sigma |
| PP-1 analog | 567809 | 4 μM | Calciochem |
| MK2206 |  | 10 μM | Calciochem |
| PD98059 |  |  |  |
| KN93 |  | 1 μM | Sigma |
| 2-APB |  | 10, 30, and 100 μM | Calciochem |
| Nifedipine |  | 5 μM | Sigma |
| Cyclosporine A |  | 5 μM | Sigma |
| Ca^2+^-free medium |  | 5.5 mM D-glucose, 130 mM NaCl, 5.4mM KCl, 20 mM HEPES (pH=7.4), and 3 mM MgSO_4_ |  |
| Buffered salt solution (BSS) |  | 5.5 mM D-glucose, 130 mM NaCl, 5.4mM KCl, 20 mM HEPES (pH=7.4), 1 mM MgSO_4_, and 2 mM CaCl_2_ |  |
