# Supplementary material for: Dynamic Trk and G Protein Signalings Regulate Dopaminergic Neurodifferentiation in Human Trophoblast Stem Cells
Source: PLoS One. 2015 Nov 25;10(11):e0143852. doi: 10.1371/journal.pone.0143852 (PMC4659658; doi:10.1371/journal.pone.0143852)
Supplement: S2 Table — (DOCX) [file pone.0143852.s008.docx]

**S2 Table. Primary and second antibodies used in this study**

| **Primary Ab** | **For WB** | **For IP** | **For IF** | **For flow** | **Cat. no** | **Source** | **Manufacturer** |
| --- | --- | --- | --- | --- | --- | --- | --- |
| p-4EBP1  Thr70 | 1:5000 |  |  |  | 1578-1 | Rabbit | Epitomics |
| p-Akt  Ser473 | 1:1000 |  |  | 1:100 | 4051S | Mouse | Cell Signaling Technology |
| Akt1 | 1:1000 |  |  | 1:100 | 2967S | Mouse | Cell Signaling Technology |
| Akt2 | 1:1000 |  |  | 1:100 | 2964S | Rabbit | Cell Signaling Technology |
| Akt3 | 1:1000 |  |  | 1:100 | 4059S | Rabbit | Cell Signaling Technology |
| α-tubulin | 1:1000 | 1:200 |  |  | 2144 | Rabbit | Cell Signaling Technology |
|  | 1:1000 |  |  | 1:200 | ab7291 | Mouse | Abcam |
| β-tubulin | 1:200 |  |  |  | sc-5274 | Mouse | Santa Cruz biotechnology |
| Calcinurin | 1:1000 |  |  |  | 2614 | Rabbit | Cell Signaling Technology |
| CaMKII | 1:200 |  |  |  | sc-9035 | Mouse | Santa Cruz biotechnology |
| CREB1 | 1:1000 | 1:200 | 1:200 |  | 4820 | Mouse | Cell Signaling Technology |
| p-CREB ser133 | 1:1000  1:1000 | 1:40  1:150 | 1:100 |  | T3707  9196 | Rabbit  Mouse | Epitomics  Cell Signaling Technology |
| p-c-Raf  Ser338 | 1:1000 |  |  |  | 9427P | Rabbit | Cell Signaling Technology |
| c-Src | 1:150 |  |  |  | sc-166860  sc-130069 | Mouse Mouse | Santa Cruz biotechnology |
| Dvl3 | 1:500 |  |  |  | 2093-1 | Rabbit | Epitomics |
| eIF4E | 1:1000 |  |  |  | 9742S | Rabbit | Cell Signaling Technology |
| p-eIF4E  Ser209 | 1:1000  1:2000 |  |  |  | 9741  2227-1 | Rabbit | Cell Signaling Technology  Epitomics |
| elf4B | 1:1000 | 1:100 |  |  | 3592S | Rabbit | Cell Signaling Technology |
| EP300 | 1:200 |  |  |  | sc-56455 | Mouse | Santa Cruz biotechnology |
| Erk | 1:1000 |  |  |  | 4695 | Rabbit | Cell Signaling Technology |
| p-Erk | 1:1000 |  |  |  | 4377 | Rabbit | Cell Signaling Technology |
| FRAT1 | 1:1000 |  |  | 1:100 | 3215-1 | Rabbit | Epitomics |
| Fzd6 |  |  |  | 1:100 | SAB4503267 | Rabbit | Sigma-Aldrich |
| GSK3β | 1:200 |  |  |  | ab18893 | Rabbit | Abcam |
| GSK3α/β  Ser9/21 | 1:1000 | 1:100 |  | 1:100 | 9331 | Rabbit | Cell Signaling Technology |
| GSK3α/β  Tyr279/216 | 1:1000  1:1000 |  |  | 1:100 | 05-413  GM1321 | 05-413  Mouse | EMD Millipore  ECM biosciences |
| Gαq/11 | 1:250 |  | 1:100 | 1:100 | sc-46972 | Rabbit | Santa Cruz biotechnology |
| Gβ | 1:150 |  | 1:100 |  | sc-166064 | Mouse | Santa Cruz biotechnology |
| Importin | 1:200 |  |  |  | sc-365231 | Mouse | Santa Cruz biotechnology |
| IP3R | 1:500 |  |  |  | 07-1210 | Rabbit | EMD Millipore |
| Lamin A/C | 1:1000 |  |  |  | 2032S | Rabbit | Cell Signaling Technology |
| LEF1 | 1:1000 |  |  |  | 2458-1 | Rabbit | Epitomics |
| MAPT | 1:200 | 1:50 |  | 1:50 | sc-32274 | Mouse | Santa Cruz biotechnology |
| MEF2A | 1:1000  1:500 | 1:250 |  | 1:200 | ab76063  9736S | Rabbit | Abcam  Cell Signaling Technology |
| p-Mek  Ser217/221 | 1:1000 |  |  |  | 9154 | Rabbit | Cell Signaling Technology |
| mTOR | 1:1000 | 1:100 |  |  | 2983 | Rabbit | Cell Signaling Technology |
| p-mTOR Ser2448 | 1:1000 |  |  |  | 2971 | Rabbit | Cell Signaling Technology |
| Nanog | 1:200  1:500 |  |  |  | sc-30332  AB9220 | Goat  Rabbit | Santa Cruz biotechnology  EMD Millipore |
| N-cadherin | 1:1000 | 1:100 |  | 1:80 | 4061 | Rabbit | Cell Signaling Technology |
| NFAT1 | 1:1000 | 1:200 |  | 1:100 | 5862 | Rabbit | Cell Signaling Technology |
| Parkin | 1:200 | 1:50 |  | 1:80 | 2132 | Mouse | Santa Cruz biotechnology |
| PI3K | 1:200 |  |  |  | sc-7174 | Rabbit | Santa Cruz biotechnology |
| PIP2 | 1:150 |  |  |  | sc-53412 | Mouse | Santa Cruz biotechnology |
| Pitx2 | 1:1000  1:100 | 1:200 | 1:100 |  | ab55599  sc-8748 | Rabbit  Goat | Abcam  Santa Cruz biotechnology |
| Pitx3 | 1:100 |  |  |  | sc-19307 | Goat | Santa Cruz biotechnology |
| p-PKA  Thr197 | 1:1000 |  |  |  | 5661 | Rabbit | Cell Signaling Technology |
| RARβ | 1:200 |  | 1:100 |  | sc-552 | Rabbit | Santa Cruz biotechnology |
| RXRα | 1:200 |  | 1:80 |  | sc-46659 | Mouse | Santa Cruz biotechnology |
| α-synuclein | 1:200 | 1:50 | 1:80 |  | sc-12767 | Mouse | Santa Cruz biotechnology |
| Stat3 | 1:200 |  |  |  | sc-8019 | Mouse | Santa Cruz biotechnology |
| p-Stat3  Ser727 | 1:800 |  |  |  | 9134 | Rabbit | Cell Signaling Technology |
| TH | 1:100 | 1:50 | 1:80 |  | sc-14007 | Rabbit | Santa Cruz biotechnology |
| WNT2B | 1:1000 | 1:250 |  |  | ab178418 | Rabbit | Abcam |
| β-catenin | 1:1000 | 1:100  1:200 | 1:200  1:200 | 1:100 | 8480  ab16051 | Rabbit  Rabbit | Cell Signaling Technology  Abcam |
| β-actin | 1:2000  1:2000 |  |  |  | sc-47778  4970 | Mouse  Rabbit | Santa Cruz biotechnology  Cell Signaling Technology |

Abbreviation: WB: Western blot; IP: Immunoprecipitation; IF: immunofluorescence; Flow: Flow cytometry.
