# Supplementary material for: Dynamic Trk and G Protein Signalings Regulate Dopaminergic Neurodifferentiation in Human Trophoblast Stem Cells
Source: PLoS One. 2015 Nov 25;10(11):e0143852. doi: 10.1371/journal.pone.0143852 (PMC4659658; doi:10.1371/journal.pone.0143852)
Supplement: S3 Table — (DOCX) [file pone.0143852.s009.docx]

**S3 Table. siRNAs used in this study**

| **siRNA target** | **Cat. No.** | **Manufacturer** |
| --- | --- | --- |
| Scrambled | [#AM4611](http://www.ambion.com/search/click_log_filter.php?link=http%3A%2F%2Fwww.ambion.com%2Fcatalog%2FCatNum.php%3FAM4611&stl=2000377&p=2147) | Ambion |
|  | 0007786733-1 | Sigma-Aldrich |
|  | TRCN0000072178 | National RNAi Core Facility Platform |
| Akt1 | SASI_Hs01_00205545 | Sigma-Aldrich |
| Akt2 | SASI_Hs01_00035058 | Sigma-Aldrich |
| Akt3 | SASI_Hs01_00122808 | Sigma-Aldrich |
|  | TRCN0000001615  TRCN0000001616 | National RNAi Core Facility Platform |
| CREB1 | SASI_Hs01_00116985 | Sigma-Aldrich |
|  | TRCN0000011085  TRCN0000226467  TRCN0000226468  TRCN0000007310 | National RNAi Core Facility Platform |
| eIF4E | SASI_Hs01_00216117 | Sigma-Aldrich |
|  | TRCN0000062575  TRCN0000062576  TRCN0000062573 | National RNAi Core Facility Platform |
| eIF4B | SASI_Hs01_00121426 | Sigma-Aldrich |
|  | TRCN0000236423  TRCN0000236424  TRCN0000062598 | National RNAi Core Facility Platform |
| 4EBP1 | SASI_Hs02_00336903 | Sigma-Aldrich |
| Gβ | TRCN0000036779  TRCN0000036780  TRCN0000036783 | National RNAi Core Facility Platform |
| mTOR | SASI_Hs02_00338641 | Sigma-Aldrich |
|  | 6381 | Cell signaling |
|  | TRCN0000195453  TRCN0000199323 | National RNAi Core Facility Platform |
| NFAT1 | SASI_Hs01_00195473 | Sigma-Aldrich |
| MEF2A | SASI_Hs01_00163355 | Sigma-Aldrich |
|  | TRCN0000005132  TRCN0000005134 | National RNAi Core Facility Platform |
| Pitx2 | TRCN0000020479  TRCN0000020481  TRCN0000020483 | National RNAi Core Facility Platform |
| PI 3K p101 | SC-94221 | Santa Cruz |
| FRAT1 | SASI_Hs01_00129296 | Sigma-Aldrich |
| Fzd6 | SASI_Hs02_00335793 | Sigma-Aldrich |
| RARβ | SASI_Hs01_00062416 | Sigma-Aldrich |
| RXR | SASI_Hs01_00097638 | Sigma-Aldrich |
| Stat3 | SASI_Hs01_00121206 | Sigma-Aldrich |
| TrkA | TRCN0000001992  TRCN0000001995  TRCN0000199815 | National RNAi Core Facility Platform |
| TrkB | TRCN0000002245  TRCN0000195539  TRCN0000196299 | National RNAi Core Facility Platform |
| TrkC | TRCN0000002309  TRCN0000002313  TRCN0000194821 | National RNAi Core Facility Platform |
| c-Src | SASI_Hs01_00112907 | Sigma-Aldrich |
| Wnt2B | SASI_Hs01_00125584 | Sigma-Aldrich |
