# Supplementary material for: Dynamic Trk and G Protein Signalings Regulate Dopaminergic Neurodifferentiation in Human Trophoblast Stem Cells
Source: PLoS One. 2015 Nov 25;10(11):e0143852. doi: 10.1371/journal.pone.0143852 (PMC4659658; doi:10.1371/journal.pone.0143852)
Supplement: S4 Table — (DOCX) [file pone.0143852.s010.docx]

**S4 Table. Primers used in this study**

| Target gene | Forward primer sequence | Reverse primer sequence |
| --- | --- | --- |
| RXRα | F: AGTCCTCAGGCTACCACTAT | R: CCTCCTTCTTCTTGTTT |
| RARβ | F: ATGGATGTTCTGTCAGTGAG | R: CATAGTGGTACCCTGATGAT |
| c-Src | F: AAGCTGTTCGGAGGCTTCAA | R: TTGGAGTAGTAGGCCACCAG |
| Fzd6 | F: AAGCGGACGGAGCTAGCACC | R: GTTGCTCTGTGCCAGGCCAG |
| Dvl3 | F: CTCCACCAGCTCCTCCATCAC | R: CTGGTACGGGAAAGCCATGGG |
| FRAT1 | F: GCCCTGTCTAAAGTATTTTCAG | R: CGCTTGAGTAGGACTGCAGAG |
| GSK3β | F: CCGGAGGCAATTGCACTGTG | R: AGGATGGTAGCCAGAGGTGG |
| β-actin | F: GTGGGGCGCCCCAGGCACCA | R: CTCCTTAATGTCACGCACGATTTC |
| β-catenin | F: GGTAGGGTGGGAGTGGTTTAGGC | R: ATACCAGCCCACCCCTCGAGC |
| Akt1 | F: GGACGCCAAGGAGATCATGC | R: CGCTGTCCACACACTCCATG |
| Akt2 | F: GGCCCCTGATCAGACTCTA | R: TCCTCAGTCGTGGAGGAGT |
| Akt3 | F: GCAAGTGGACGAGAATAAGTCTC | R: ACAATGGTGGGCTCATGACTTCC |
| Nurr1 | F: CTGTAACTCGGCTGAAGCCAT | R: AGTGTTGGTGAGGTCCATGC |
| ALDH1A1 | F: ATGCCGACTTGGACAATGCT | R: ACAAAAATCCTGGATGCGGC |
| GIRK2 | F: GAGCAGAATGGAGTCTCCTAACA | R: GCTTTTCCTGGCTGCGGAC |
