# Supplementary material for: Dynamic Trk and G Protein Signalings Regulate Dopaminergic Neurodifferentiation in Human Trophoblast Stem Cells
Source: PLoS One. 2015 Nov 25;10(11):e0143852. doi: 10.1371/journal.pone.0143852 (PMC4659658; doi:10.1371/journal.pone.0143852)
Supplement: S1 Text — (DOCX) [file pone.0143852.s011.docx]

Supporting Information

**Materials and Methods**

**Cell Culture and Differentiation**

hTS cells obtained from the preimplantation embryos in women with early tubal ectopic pregnancy were described previously [5]. After two passages, the level of hCG became undetectable measured by a commercial kit (Dako, Carpinteria, CA). Adherent hTS cells were cultured in conditioned *α*-MEM plus 10% FBS at 37^o^C in 5% CO_2_. For neural cell differentiation, cells at passages between 5 and 8 in cultivation were used for neurogenic differentiation by treatment with all-*trans* retinoic acid (10 μM) (RA; Sigma-Aldrich).

**Western Blot**

Cells were lysed in RIPA buffer containing a protease inhibitor cocktail. Protein concentrations were determined by the BCA method. Equal amounts of proteins were resolved on 6%–12% (w/v) SDS-PAGE and transferred onto polyvinylidene fluoride membranes. After blocking with nonfat dried milk, the membranes were incubated with primary antibodies listed in Table S1 overnight at 4°C, followed by detection using horseradish peroxidase–labeled secondary antibodies. The proteins were visualized by immobilon Western Chemiluminescent HRP Substrate (Millipore). The proteins were stripped from the blotting membrane by incubation in Restore PLUS Western Blot stripping buffer (Thermo Scientific).

**Immunoprecipitation (IP)**

Cells were serum-deprived overnight and treated with RA (10 μM) for 4 hr or 24 hr as indicated. Cells were lysed by RIPA lysis buffer (Millipore). The mixtures of lysate and protein A or protein G agarose (Minipore) were incubated with rocking at 4^0^C for 2 hr. Specific primary antibody or rabbit IgG (control) was added and incubated overnight. The immune protein complex was then captured on beads with either protein A or protein G. The antibody-bound proteins were precipitated by rocking for overnight. The immunoprecipitated proteins were washed with RIPA lysis buffer followed by analysis with SDS-PAGE and immunoblotting with another specific antibody to measure the interaction.

**RNA Interference**

Small interfering RNA (siRNA) and short hairpin RNA (shRNA) used were purchased from the National RNAi Core Facility Platform, Institute of Molecular Biology/Genomic Research Center, Academia Sinica, Taipei, Taiwan and listed in Table S2. Transfection was performed using TransIT®-LT1 transfection reagent (Mirus, Madison, WI, USA) at a 2:1 (LT1:DNA) charge ratio in Opti MEM medium (Life Technologies, Grand Island, NY). Cells were assayed after overnight transfection. The scrambled siRNA or shGFP (not targeting any human gene) was used as control.

**Flow Cytometry**

Method used was described as previously^4^. Cells (5 × 10^6^ cells/ml) were incubated with a variety of primary antibodies for 30 min and then incubated with the appropriate fluorescein isothiocyanate (FITC)-, phycoerythrin (PE)- or Rho-conjugated secondary antibody (Jackson ImmunoResearch, West Grove, PA) at adjusted dilution for 1 hr at 4^o^C. After thorough washing, cells were re-suspended in PBS (1 ml) and subjected to flow cytometry (FACScan, BD Biosciences, San Jose, CA). The data were analyzed with Cell-Quest software (BD Biosciences).

**Immunocytochemistry and TissueQuest Analysis**

For hTS cells, cells were cultured on the Lab-Tek™ chamber slide (Nalge Nunc International, Naperville). Cells were fixed in ice-cold methanol:acetone (1:1) at -20°C for 10 min and blocking by blocking buffer (1% BSA in PBS) for 1 hr at 37°C. Chamber slides were incubated with the diluted primary antibody as indicated (listed in Table S1) overnight at 4°C, followed by adding fluorescent conjugated secondary antibody for 1 hr at room temperature. After PBS washing, cells were counterstained by 4',6-diamidino-2-phenylindole (DAPI) and sealed with coverslip for microscopy.

For brain tissue, coronal brain sections (30 μm) were permeabilized with 0.2% Triton X-100 in PBS (30 min). After blocking with 5% BSA (20 min) at room temperature, cells were incubated with monoclonal antibody against TH (1:100; Santa Cruz biotechnology) or CREB-1 (1:200; Cell Signaling Technology) for 24 h at 4°C. After staining with Texas red- or FITC-conjugated secondary antibodies for 1 hr at room temperature, samples were observed by Olympus FluoView 1000 confocal laser scanning microscope or Zeiss AxioImager Z1 microscope. Data were further processed by TissueFaxs software (TissueGnostics, Vienna, Austria). For quantitative analysis, TH (+) CREB-1(+) DA neurons in the lesioned substantia nigra compacta (SNC) side were counted compared to the normal one. Cells with bizarre size or intensity outside the normal (e.g., artifact or unusually heavy stain area or intensity) were excluded. Data were analyzed by two technicians independently.

**Double Immunogold Electron Microscopy**

Cells were treated by RA (10 μM) in the dark for 15 min followed by wash with iced PBS. After incubation with 4% glutaraldhyde (6 ml) in microwave at 4^o^C for 10 min, cells were moved to an eppendorf and centrifuged at 3,000 rpm for 3 min to remove the supernatant. By adding 1% osmium tetroxide (200 μl) into eppendorf and moved to the microwave again for 1 min. After centrifugation (3,000 rpm, 3 min), the cells were treated with 50, 70, 90, and 100% ETOH each at 37^o^C for 1 min followed by adding LR White embedding medium (1:1 v/v) to maintain at 45^o^C for 15 min. Cells were incubated with 100% LR White embedding medium at 45^o^C (15 min), 60^o^C (10 min), 70^o^C (10 min) and 80^o^C (25 min). Ultrathin sections (70–80 nm) were prepared with an ultramicrotome (Reichert-Jung) and treated by 0.01M sodium citrate (pH 6.0, 1 ml) in autoclave (121^o^C, 1.5 atm) for 15 min. These fixed ultrathin sections were pretreated with an aqueous solution of 5% sodium metaperiodate (10 min) and washed with distilled water. Grids incubated with an aliquot of IgG antibody against RXRα (1:50, Santa Cruz) or Gα_q/11_ (C-19; sc-392; 1:50, Santa Cruz) and followed by probing with a secondary anti-mouse IgG 6 nm gold particles (1:10; AB Chem, Dorval, Canada) or anti-rabbit IgG 20 nm gold particles (1:10, BB International, UK). Grids were washed with PBS between incubation steps and sections blocked by placing the grids on a drop of PBS with 1% ovalbumin (15 min). After IgG gold, the grids were jet-washed with PBS followed by distilled water. All steps were carried out at room temperature. Sections were then stained with uranyl acetate and lead citrate and characterized on a Hitachi H-700 model transmission electron microscopy (JEOL JEM-1200EX, Hitachi Ltd., Japan) at 100KV. Some grids were not treated with sodium metaperiodate, which were stained in the similar way for comparative purposes and used as controls.

**Live Cell Imaging and Calcium Measurements**

For live cell imaging studies, cells were seeded onto coverslips and incubated in serum-free medium overnight. For intracellular calcium signaling detection, cells were pretreated with a variety of chemicals, including KCl, nifedipine, and 2-APB for 30 min. Then cells were loaded with Fura-2, a Ca^2+^-specific dye, in HBSS buffer at room temperature for 20 min to measure the calcium responses. Ca^2+^ free medium contains EGTA (1.2 mM/L; Applichem) and Thapsigargin (10 mM/L; Calbiochem). Intracellular calcium responses were analyzed by real-time cell imaging microscopy (Olympus, Cell-R) and Olympus Cell-R imaging software.

**Quantitative PCR (qPCR)**

Total RNA was extracted using TRIZOL reagent (Invitrogen) and mRNA expression by using a Ready-To-Go RT-PCR beads kit (Amersham Biosciences, Buckinghamshire, UK). RNAs were extracted from hTS cells by Trizol as described (Invitrogen). Briefly, concentration of RNA was measured by Epoch spectrophotometer (BioTek). RNA was suspended in the nuclease-free water and stored at -80 until used. cDNA was prepared from RNA (1 μg) using reverse transcription system (Promega) and oligo (dT) as primer according to the manufacturer's instructions. Quantitative PCR was conducted to the cDNA (1 μl) which was amplified in triplicate or more using SYBR Green PCR Master Mix (Applied Biosystems). Primers using listed in Table S3. Gene expression was normalized to the level of *Gapdh* or *β-actin* expression.

**ChIP-qPCR Assay**

ChIP assay was performed by using chromatin immunoprecipitation kit (Active motif, Carlsbad, CA) according to the instructions. Briefly, hTS cells were added with 1% formaldehyde to crosslink protein to DNA and was stopped the reaction by glycine. After centrifugation, pellets were resuspended in SDS lysis buffer with protease inhibitor. The nuclei were sheared by enzymatic shearing kit to an average size of 500 bp. The crosslinked chromatin was incubated with anti-RNA Polymerase II (positive control) or normal mouse IgG (negative control) or specific primary antibody at 4°C overnight. The immune complex was washed with ChIP buffer and the protein/DNA complexes were resuspended in elution buffer AM2. Chromatin was incubated with reverse cross-linking buffer to reverse the DNA-protein crosslink, followed by removing the contaminated RNA and protein using RNase A and proteinase K. Immunoprecipitated DNA was collected by spin filter and subjected for qPCR using specific promoter primer sets as listed in Table S4 or EpiTect-ChIP-qPCR primers (Qiagen Inc., Valencia, CA), corresponding to the human gene promoter region. qPCR product was performed on a 2% agarose gel stained with 0.01 % ethidium bromide.
